# Lands’ Cycle at the Crossroads: Phospholipid Remodelling, Oxidative Stress, Cellular Toxicity, and Therapeutic Targeting

**DOI:** 10.1021/acsptsci.5c00482

**Published:** 2025-11-03

**Authors:** Airam Roggero, Marcos H. Toyama, Sergio F. Sousa

**Affiliations:** † LAQV/REQUIMTE—BioSIM, Department of Biomedicine, 26706 Medicine Faculty of Porto University, Porto District, Porto 4099-002, Portugal; ‡ BIOMOLPEP, Department of Biology, Institute of Biosciences UNESP/CLP, São Paulo 11330-900, São Vicente, Brazil; § Department of Chemical Engineering, Faculty of Science and Technology, University of Coimbra, Coimbra 3004-531, Portugal

**Keywords:** lipid metabolism, phospholipases, acyl transferases, membrane remodelling, oncogenesis

## Abstract

The Lands cycle is a fundamental process for the continuous renewal of phospholipids in cell membranes, directly influencing their fluidity and functionality. This cycle is particularly active in tissues such as the nervous and immune systems and is crucial for cellular homeostasis. It is implicated in the development of inflammatory, neurodegenerative, and cancerous diseases. The present review discusses the biochemical regulation of the Lands cycle—focusing on phospholipase A2 (PLA_2_) and lysophospholipid acyltransferase (LPCAT)—and its impact on lipid metabolism, cell signaling, and disease. Dysregulation of this cycle has been linked to pathological conditions, including oncogenesis and hepatotoxicity. This suggests that modulation of the cycle may have an effect on inflammatory responses and tumor resistance. Advances in the fields of lipidomics and computational modeling have resulted in a more comprehensive understanding of the Lands cycle, thereby emphasizing its potential as a therapeutic target.

## Graphical abstract

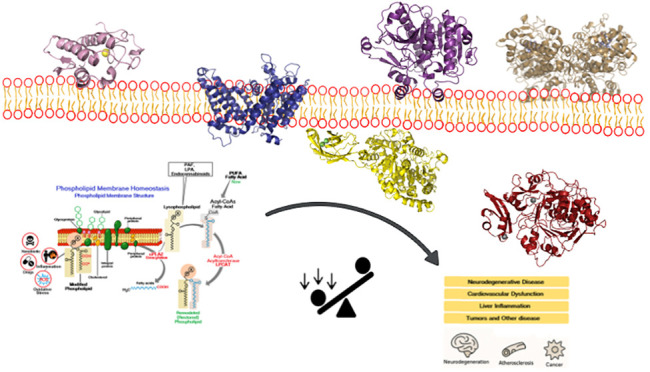

The Lands cycle, first described by John Lands in 1960, is essential for the ongoing regeneration of membrane phospholipids, thereby maintaining membrane fluidity and cellular adaptability. This process is of particular significance in tissues exposed to environmental fluctuations, such as the nervous and immune systems. Cell membranes, which are composed primarily of a phospholipid bilayer and proteins, are dynamic structures. The integrity and functionality of these membranes depend on the precise regulation of their lipid components.^1,2^

Phospholipids constitute the primary components of biological membranes, with glycerophospholipids, including phosphatidylcholine (PC), phosphatidylethanolamine (PE), phosphatidylserine (PS), phosphatidylinositol (PI), and phosphatidic acid (PA), representing the predominant lipids involved in the structural organization of mammalian membranes. Phosphatidylcholine (PC) is the most abundant of the glycerophospholipids, representing approximately 40–50% of the total phospholipids in mammalian membranes and subcellular organelles. PC is synthesized by the Kennedy pathway and undergoes extensive remodelling via the Lands cycle.^3–5^

The Lands cycle represents a process that is responsible for the continuous turnover of phospholipids. This process involves the cleavage of these lipids by phospholipase A2 (PLA_2_) and the reacylation of lysophospholipids by lysophosphatidylcholine acyl transferase (LPCAT).^4,6^ The maintenance of this cycle is essential for the stability of biological membranes, the formation of lipid droplets, the regulation of lipid metabolism and cell signaling.^7^

It has been demonstrated that alterations in the Land cycle are associated with a range of diseases. In the context of inflammation, alterations in membrane lipid composition have been demonstrated to modulate receptor activation, as the Toll-like receptors (TLRs) and the subsequent generation of pro-inflammatory mediators, including eicosanoids. In the context of cancer, the overexpression or dysregulation of PLA_2_ and LPCAT has been demonstrated to induce alterations in membrane dynamics, thereby promoting tumor cell proliferation, invasion, and resistance to apoptosis and therapy. In the liver, disruptions to the cycle compromise membrane integrity, exacerbating hepatotoxicity and inflammatory responses.^8,9^

This remodelling enables cells to adjust their lipid composition in response to developmental, metabolic, and environmental cues, influencing processes such as signaling, transport, and inflammation. The cycle comprises two primary enzymatic steps. First, phospholipids are cleaved by PLA_2_ at the sn-2 position, resulting in lysophospholipids and free fatty acids. Second, lysophospholipids are reacylated by LPCAT, each exhibiting distinct tissue distributions and substrate specificities, coordinate these reactions in order to maintain membrane homeostasis. The release of arachidonic acid (AA) by the action of PLA_2_ and its conversion into pro-inflammatory eicosanoids, such as prostaglandins and leukotrienes, also plays a significant role in the amplification of inflammation. Alterations in the Lands cycle have been linked to a number of pathological conditions, including inflammatory, neurodegenerative, and cardiovascular diseases, as well as oncogenesis and hepatotoxicity.^10,11^

Recent studies have indicated the potential for therapeutic intervention targeting the Lands cycle, both through the pharmacological modulation of its enzymes and via lifestyle factors such as diet and exercise. It has been demonstrated that the modulation of the cycle can exert an influence on the resistance of cancer cells to apoptosis, thereby promoting tumor progression and resistance to programmed cell death. In the liver, the Lands cycle exerts a direct influence on liver health.^12,13^

The continuous turnover of phospholipids in cell membranes is essential for cells to respond effectively to environmental changes and external signals. This process is particularly important during embryonic development, when rapid cell division and differentiation require constant remodelling of membranes. The ability to modulate the lipid composition of membranes is therefore essential for adaptation to different environmental conditions and for maintaining homeostasis in vertebrates. In mammals, the Lands cycle plays a crucial role in cellular homeostasis and metabolic development, particularly in liver tissue, which is highly dynamic and capable of regeneration.^14,15^

Biochemically, the Lands cycle involves two main enzymatic mechanisms for the continuous renewal of cell membrane phospholipids: phospholipid cleavage and reacylation, as we can see in the Figure 1. These processes ensure the maintenance of membrane fluidity and cellular adaptability. There are several isoforms of PLA_2_ involved in this cycle, such as secreted (sPLA_2_), cytosolic (cPLA_2_), lipoprotein-associated (Lp-PLA_2_ or PAF AH) and calcium-dependent (iPLA_2_), each with different substrate specificities and functions.^15,16^

**Figure 1:**
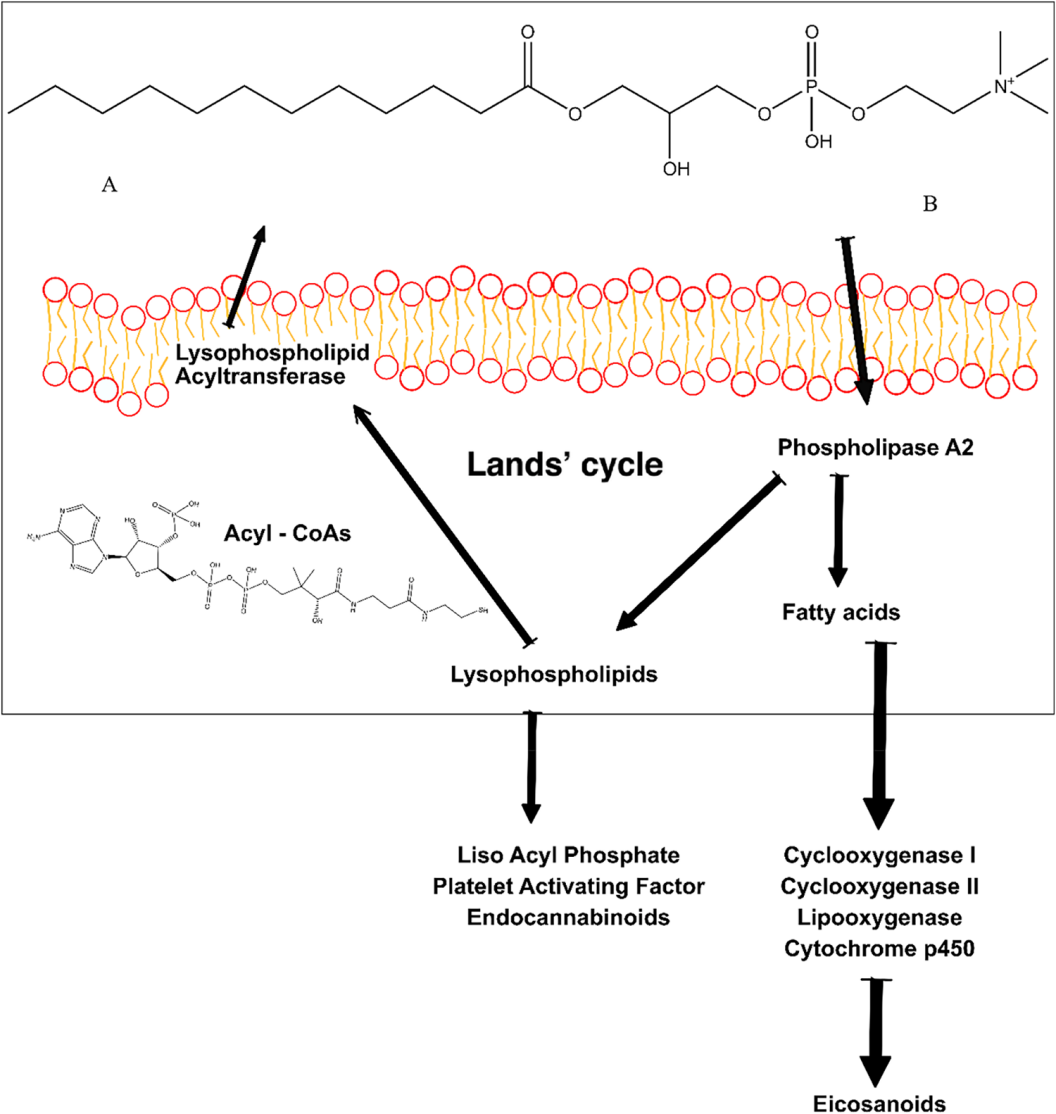
Lands cycle, divided into two main steps. Figure 2A—lysophospholipids reacylation. Figure 2B—phospholipid cleavage.

PLA_2_ activity is regulated by factors such as calcium concentration, pH and post-translational modifications. The released fatty acid can serve as a precursor for decanoic synthesis, making this process critical for membrane remodelling by allowing regeneration of phospholipids and modulation of their biophysical properties.^17,18^

In the first step of the Lands cycle, mediated by PLA_2_, phospholipids are cleaved at the sn-2 position, removing a fatty acid and forming lysophospholipids (LPL) and free, often unsaturated, fatty acids. This process is essential for the regeneration of phospholipids and the modulation of the biophysical properties of the membrane, allowing the continuous remodelling required for cellular functionality.^19,20^

The second step, mediated by LPCAT, involves the reacylation of lysophospholipids generated by the action of PLA_2_. LPCAT transfers a fatty acid from an acyl-CoA molecule to the lysophospholipids, restoring a complete phospholipid and hence the membrane structure. There are several isoforms of LPCAT (LPCAT1, LPCAT2, LPCAT3 and LPCAT4), which differ in substrate specificity and tissue expression.^21,22^

It is evident that LPCAT activity, notably that of LPCAT1 and LPCAT3, is associated with the acyltransferase family within the Lands remodelling pathway. Notably they fulfill a pivotal function in preserving the composition and fluidity of cell membranes through the process of reacylation of LPC into PC.^1^ It is notable that both enzymes possess an HXXXXD catalytic domain, a hallmark of the membrane-bound O-acyltransferase (MBOAT) superfamily. This domain facilitates their functional integration into endoplasmic reticulum membranes^23^ LPCAT3 is found in high concentrations in various tissues, including the liver, small intestine, and adipocytes. It has been demonstrated to facilitate the preferential insertion of PUFAs, such as linoleic acid and AA, into membrane PCs.^24,25^

This characteristic confers on LPCAT3 a pivotal role in sensitivity to ferroptosis, as the presence of self-oxidizing PUFAs promotes lipid peroxidation in states of glutathione peroxidase 4 (GPX4) deficiency.^26–29^ In contrast, LPCAT1 is predominantly expressed in the lungs, erythrocytes, and sebaceous glands, where it is responsible for the biosynthesis of dipalmitoylphosphatidylcholine (DPPC), the primary component of pulmonary surfactant. In addition, LPCAT1 overexpression has been linked to tumor progression by promoting plasma membrane reorganization, resistance to oxidative stress, and enhanced proliferative capacity.^30–32^

Despite the presence of analogous catalytic properties, LPCAT3 and LPCAT1 fulfill different roles in the cellular response to oxidative stress. It has been hypothesized that LPCAT3 functions as a potential sensitizer to ferroptosis, while LPCAT1 exerts a protective structural effect, depending on the tissue and redox context.^27,33^ Research conducted on the deletion of LPCAT3 has revealed the intracellular accumulation of LPC, endoplasmic reticulum dysfunction and the activation of adaptive responses to stress. These effects are mitigated to a certain extent by LPCAT1, yet the incorporation profile of unsaturated fatty acids remains incomplete. Consequently, the LPCAT3–GPX4–iPLA_2_ axis is identified as a pivotal regulator of the equilibrium between repair and lipid death in iron-dependent processes. This positions these enzymes as significant targets for the development of therapies for cancer, neurodegenerative diseases, and inflammatory disorders.^26,28,34,35^

The enzymes PLA_2_ and LPCAT are critical to the cycle and their activity is finely regulated to meet the needs of the cell. The Lands cycle is essential for the maintenance of cellular homeostasis, response to external stimuli and important physiological processes such as development and inflammation.

Alterations in LPCAT function in pathological conditions such as chronic inflammation and metabolic diseases have significant implications for cellular physiology and the progression of diseases such as atherosclerosis and liver disease.^36^

Precise regulation of the Lands cycle is crucial for the maintenance of neuronal function and the adaptive response of the immune system. Dysfunctions in the activity of PLA_2_ and LPCAT can lead to significant metabolic disorders and contribute to the development of cell membrane-related diseases.^37^ During embryonic development, the fluidity of cell membranes is essential for intercellular signaling and communication. The coordinated activity of PLA_2_ and LPCAT ensures that phospholipids are continually recycled and replenished, allowing cells to maintain their functionality and integrity, particularly in rapidly expanding and differentiating tissues such as the developing nervous system.^38,39^

Research indicates that the Lands cycle is critical for metabolic development in zebrafish, directly influencing cell membrane composition and cell signaling. Animal studies show that dysfunctions in this cycle are associated with several pathological conditions, such as neurodegenerative diseases, chronic inflammation and metabolic disorders.^25,40^

In oncogenesis, Lands cycle enzymes play a crucial role by affecting the composition and fluidity of cell membranes, thereby facilitating the proliferation, invasion, and metastasis of cancer cells. Dysregulation of these enzymes can alter membrane dynamics, impacting cell signaling and allowing cancer cells to rapidly adapt to changes in their microenvironment, contributing to tumor progression and evasion of apoptosis. Overexpression of PLA_2_ is observed in various types of cancer and is associated with poorer prognosis, suggesting that this enzyme promotes a pro-inflammatory environment that facilitates the survival and proliferation of malignant cells.^41,42^

In addition, dysregulation of acyl transferases can alter the diversity and fatty acid composition of membrane phospholipids, modifying membrane fluidity and cell signaling. These changes can influence the resistance of tumor cells to chemotherapeutic drugs by altering drug uptake and efflux, as well as facilitating processes such as cell migration and invasion into adjacent tissues, which are essential for tumor progression and metastasis.^41,43^

The use of these enzymes as potential biomarkers may aid in the early diagnosis and prognosis of cancer, providing a more personalized and effective approach to treatment. The development of PLA_2_ inhibitors or modulators of acyl transferase activity represents a new therapeutic frontier, with the potential to limit cancer growth and spread in a less aggressive and more targeted manner.

Dysfunctions in this cycle can compromise the integrity of hepatocyte membranes, leading to the release of inflammatory mediators and exacerbating liver damage. Drug-induced hepatotoxicity (DILI) represents a significant cause of liver failure and is associated with alterations in the Lands cycle. This suggests that modulation of the cycle may represent a promising therapeutic strategy.^3,44^

The Lands cycle represents a crucial interface between the biochemistry of cell membranes and the pathophysiology of numerous diseases, emerging as a potential target for therapeutic interventions. New research points to alternative therapeutic approaches, such as the influence of diet and physical exercise on the regulation of this cycle. Advancements in techniques such as lipidomics and computational modeling are deepening the understanding of molecular mechanisms and their relationship with several pathological conditions.^3,12^

In addition to PLA_2_, phospholipase B (PLB) plays a significant role in lipid homeostasis and inflammation, contributing to the modulation of cell membrane composition. PLB is capable of hydrolyzing both the sn-1 and sn-2 positions of phospholipids, which influences the formation of lysophospholipids and has the potential to exacerbate inflammatory processes when deregulated.^44^

It is imperative to acknowledge the pivotal role of the Lands cycle during embryonic development. This dynamic process ensures the maintenance of membrane fluidity and functionality in rapidly proliferating and differentiating tissues, such as the nervous system. The capacity for phospholipids to undergo continuous remodelling enables cells to respond effectively to external signals and maintain metabolic homeostasis, particularly in dynamic organs such as the liver. Dysfunctions in this cycle have been demonstrated to be associated with metabolic and neurodegenerative diseases, as evidenced in animal models.^41,45,46^

## Integrity and Homeostatis of the Phospholipid Membrane

Maintaining the composition and structural organization of phospholipid membranes is fundamental to ensuring cellular integrity, environmental sensing, intracellular signaling and the dynamic regulation of physiological processes. Biological membranes are not simply passive barriers, but complex, dynamic structures composed primarily of a phospholipid bilayer integrated with proteins, cholesterol, and carbohydrate chains.^47,48^

Membrane fluidity, asymmetry, and curvature are tightly regulated by the type and saturation of fatty acid chains within phospholipids. The asymmetric distribution of phosphatidylserine (PS), enriched in the inner leaflet and externalised during apoptosis, is maintained by ATP-dependent flippases and ensures proper cellular signaling. These properties are critical for several cellular functions, including vesicle trafficking, membrane fusion, protein localization, and receptor activation.^49^

Over time or under physiological challenges such as oxidative stress, membrane phospholipids can undergo peroxidation or hydrolysis. These perturbations compromise barrier function, leading to loss of cellular homeostasis and activation of cell death pathways such as apoptosis.^50,51^

This is particularly evident in tissues with high metabolic or signaling demands, such as the nervous system, liver, and immune cells, where membrane adaptability is critical for survival and function.^52,53^

To maintain membrane integrity, cells have evolved sophisticated lipid remodelling systems that allow rapid turnover, repair and recycling damaged or suboptimal phospholipids. Among these systems, the Lands cycle plays a leading role in the diacylation-reacylation of membrane glycerophospholipids. Through the concerted action of PLA_2_ isoforms and lysophospholipids acyltransferases, this cycle enables the regeneration of phospholipids with specific acyl chains, thereby adjusting membrane fluidity and function in response to cellular needs, as shown in Figure 2.^54,55^

**Figure 2:**
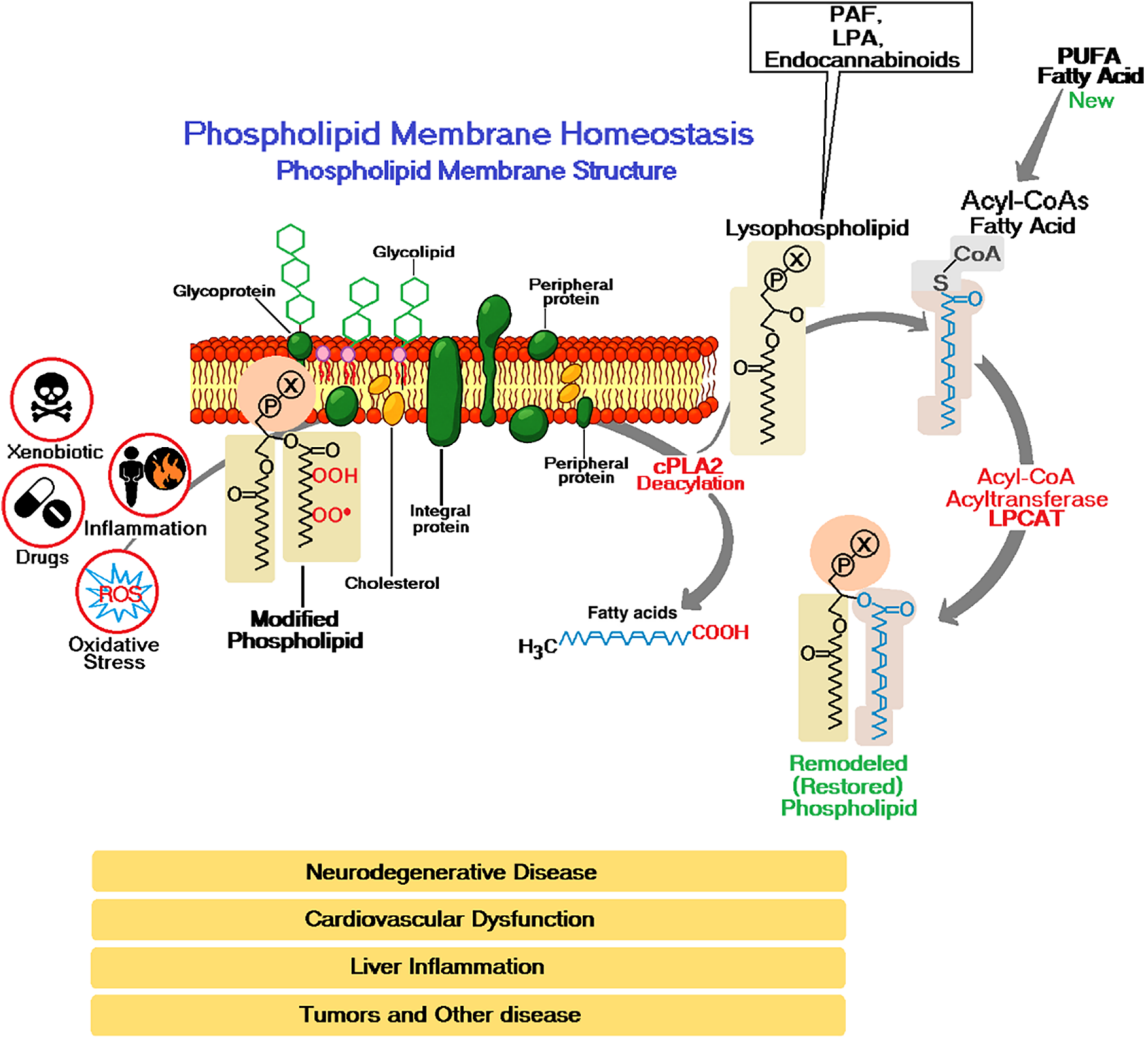
Maintenance, integrity and homeostasis of the phospholipid membrane.

Membrane repair is also critical during mechanical damage or calcium influx, such as during immune activation, ischemia, or toxin exposure. In these cases, specialized repair mechanisms involving membrane patches, endocytosis, or lipid transfer proteins are activated to reseal or replace damaged membrane segments.^51,56^

These responses are energetically demanding and require a readily available pool of phospholipid substrates, provided in part by the Lands cycle and related lipid metabolic pathways.^47,48^

The plasma membrane is a highly dynamic and functional structure composed of an asymmetric lipid bilayer enriched with integral and peripheral proteins, as well as specialized microdomains, such as lipid rafts. These domains have been demonstrated to play a pivotal role in orchestrating fundamental cellular processes, including signaling, endocytosis, and cell–cell communication.^57,58^ Membrane integrity is therefore essential for the maintenance of cellular homeostasis. However, under conditions of oxidative stress, it becomes one of the main targets of damage, especially due to the high susceptibility of PUFAs to lipid peroxidation.^59^

The process commences with the abstraction of allylic hydrogens from PUFAs, a reaction that is catalyzed by reactive oxygen species (ROS) and intensified by the presence of free ferric ions (Fe^2+^), which participate in the Fenton reaction.^60,61^ Consequently, the generation of lipid hydroperoxides (LOOHs) ensues, which swiftly undergo decomposition into reactive aldehydes, including 4-hydroxynonenal (4-HNE) and malondialdehyde (MDA). These compounds form covalent adducts with nucleophiles in proteins and phospholipids, thereby compromising membrane fluidity, selectivity, and functionality.^62,63^

The structural changes caused by this process include the disorganization of ordered lipid domains, such as lipid rafts. These domains are crucial for the recruitment of receptors, cytoskeletal anchoring proteins, and components of the endocytic machinery.^64^ The loss of these microenvironments has been demonstrated to exert a deleterious effect on signaling pathways mediated by integrins, tyrosine kinases, and GPI-anchored proteins, resulting in a profound imbalance of cellular homeostasis.^65,66^

In response to such disturbances, cells initiate complex compensatory mechanisms. A significant mechanism in this process is lipid remodelling, a process promoted by enzymes such as LPCAT3, which re-esterifies oxidized lysophospholipids, thereby restoring the functional composition of the membrane.^67,68^ This mechanism is part of the so-called Lands cycle, which is essential for the recovery of the lipid bilayer after oxidative insults. Concurrently, the neutralization of LOOHs is predominantly mediated by glutathione peroxidase 4 (GPX4), a selenoprotein that is dependent on reduced glutathione (GSH) and which converts hydroperoxides into less toxic lipid alcohols.^69^

However, failure or inhibition of these mechanisms leads to the collapse of lipid redox homeostasis and the activation of ferroptosis, a regulated form of cell death distinct from apoptosis, necrosis, and pyroptosis.^70^ Ferroptosis is characterized by the overaccumulation of iron-dependent lipid peroxides, mitochondrial dysfunction with loss of membrane potential, mitochondrial condensation, and absence of nuclear fragmentation.^71^ In contrast to the biochemical hallmarks of apoptosis, the primary feature of this process is the selective failure of lipid antioxidant defense.^72^

At the molecular level, the process of ferroptosis is subject to the regulation of multiple interconnected axes. The GPX4-GSH system is the primary barrier against peroxidation. Iron metabolism involves transporters such as transferrin, DMT1, ferroportin, and ferritin, whose expression regulates the availability of catalytically active Fe^2+^. Finally, there are transcription factors such as Nrf2 and p53, which control genes linked to GSH synthesis, cystine uptake via xCT/SLC7A11, and lipid metabolism.^73–75^

Ferroptosis has been implicated in several pathophysiological conditions. In neurodegenerative diseases, it contributes to the progressive loss of neurons vulnerable to oxidative stress; in ischemia-reperfusion injury, it exacerbates iron- and ROS-mediated tissue damage; and in cancer, it represents a promising pathway to eliminate apoptosis-resistant tumor cells, especially in hypoxic and metabolically challenging environments.^76,77^

Consequently, therapeutic modulation of ferroptosis has emerged as a relevant strategy. A number of pharmacological agents are currently being explored as potential tools to induce ferroptosis in neoplastic cells. These include GPX4 inhibitors, iron chelators and agents that promote selective iron overload in tumors. Conversely, in degenerative diseases, the preservation of GPX4 activity or the activation of the Nrf2 axis may offer neuroprotection and stabilization of cell membranes.^78,79^

In this context, natural bioactive compounds, such as flavonoids and polyphenols, as well as nonsteroidal anti-inflammatory drugs (NSAIDs), exert multifaceted effects on the integrity and dynamics of cell membranes, directly influencing compensatory mechanisms and susceptibility to ferroptosis. The NSAIDs have been shown to inhibit cyclooxygenases, thereby modulating not only inflammation but also phospholipid metabolism. This is achieved by interfering with the synthesis of eicosanoids derived from polyunsaturated fatty acids (PUFAs). This effect may impact lipid composition and membrane fluidity. Furthermore, some NSAIDs have been observed to stimulate the release of AA via PLA_2_ activation, thereby providing additional substrates for lipid peroxidation and potentially favoring ferroptosis in oxidative contexts.^80,81^

Conversely, natural compounds, including gallic acid, quercetin, and curcumin, have been shown to possess antioxidant and iron-chelating properties. These compounds have been observed to inhibit ROS formation and maintain membrane integrity by preserving the activity of enzymes such as GPX4. Furthermore, these compounds have been observed to upregulate lipid remodelling enzymes, such as LPCATs, thereby favoring the restoration of oxidized phospholipids and the maintenance of the lipid bilayer.^82,83^

Consequently, while NSAIDs have the capacity to act as dual-action agents, i.e., as both protectors and sensitizers of ferroptosis, depending on the redox and inflammatory context, natural compounds tend to reinforce compensatory membrane defense mechanisms. Consequently, they selectively modulate cellular vulnerability to ferroptosis, rendering them promising adjuvant therapeutic agents. The interface between lipid peroxidation, antioxidant response and ferroptosis therefore constitutes an emerging and highly adjustable axis for therapeutic interventions in inflammatory, neurodegenerative and oncological diseases.^80,81^

Figure 1 illustrates the molecular mechanisms involved in maintaining plasma membrane integrity through the Lands cycle, highlighting its relevance in response to cellular perturbations such as oxidative stress, inflammation, xenobiotics and drugs. These conditions can lead to the formation of oxidized or modified phospholipids, compromising the structure and function of the lipid bilayer.

The mechanisms involved in maintaining plasma membrane integrity through the Lands cycle, highlighting its relevance in responding to cellular perturbations such as oxidative stress, inflammation, xenobiotics and drugs. These conditions can lead to the formation of oxidized or modified phospholipids, compromising the structure and function of the lipid bilayer. The action of cPLA_2_ promotes the cleavage of the fatty acid from the damaged phospholipid, forming a lysophospholipid and releasing free fatty acids such as of polyunsaturated fatty acids (PUFAs).^84^

This lysophospholipid is then reacylated by LPCAT-type acyltransferases, using acyl-CoA as the donor of the new fatty acid chain, restoring the lipid composition—a key step in the Lands cycle. In addition to their structural role, lipid intermediates such as PAF, lysophosphatidic acid, and endocannabinoids are involved in cellular signaling. Failure of membrane repair can trigger or exacerbate pathological conditions such as neurodegenerative diseases, cardiovascular dysfunction, liver inflammation and cancer, as shown at the bottom of the figure. Lipid remodelling promoted by the Lands cycle is therefore essential to maintain the fluidity, selectivity and functionality of the plasma membrane.^85^

There is evidence linking disruption of membrane lipid homeostasis to a wide range of pathological conditions, including neurodegenerative diseases, cardiovascular dysfunction, liver injury, and oncogenesis.^52,53,86^ In neurodegenerative diseases such as Alzheimer’s disease, altered cholesterol metabolism disrupts membrane microdomains, impairs synaptic vesicle trafficking and promotes amyloid-β accumulation.^86^

In these contexts, phospholipid remodelling is not just a compensatory process—it becomes a driver of disease progression, either through maladaptive lipid signaling or structural membrane failure.^87,88^

In summary, it is essential to understand the architecture, maintenance and remodelling of the phospholipid bilayer before delving into the mechanistic details of the Lands cycle, which constitutes one of the most important molecular systems for maintaining membrane adaptability and cellular resilience under both physiological and pathological conditions.^54,55^

## Molecular Mechanism

The enzymes known as PLA_2_ and LPCAT have been shown to play pivotal roles in the Lands cycle. Evidently several PLA_2_ isoforms (secreted, cytosolic, lipoprotein-associated, and calcium-dependent) possess distinct specificities and are subject to regulation by factors such as calcium concentration, pH and post-translational modifications.^89,90,55,91^ ACSL (long-chain fatty acid acyl-CoA synthetase),^92^ GPX4 ^69^ and Prdx6,^93,94^ play critical roles in lipid metabolism, membrane repair and remodelling, with direct implications for modulating inflammation and maintaining liver and cardiovascular health.^95^

Subsequently, the isoforms LPCAT1 to LPCAT4 exhibit discrepancies in terms of substrate specificity and tissue expression. LPCAT3, for instance, is present in high concentrations in the liver and intestine, thus facilitating the preferential incorporation of polyunsaturated fatty acids. In contrast, LPCAT1 is predominantly expressed in the lungs and is associated with pulmonary surfactant biosynthesis and tumor progression. The coordinated activity of these enzymes is imperative for maintaining membrane fluidity and integrity, thereby influencing physiological and pathological processes.^96^ This process is part of the Lands cycle, which is essential for maintaining the integrity of cell membranes.^95^

The enzyme ACSL adds coenzyme A to the fatty acid at the sn-2 position, making it a substrate for LPCAT. This promotes the incorporation of lysophospholipids into the membrane, ensuring its repair and fluidity.^46^ Prdx6 acts in the cycling of lysophospholipids, preventing oxidative stress and the release of fatty acids that are precursors of inflammatory signaling, by avoiding interactions with amino acids that regulate the MAPK (mitogen-activated protein kinase) pathway.^94^

The MAPK pathway responds to extracellular stimuli and regulates cellular activities such as gene expression, mitosis, differentiation, cell survival and apoptosis. The enzymes Prdx6 and cPLA_2_ can promote a continuous inflammatory cycle. The enzymes cyclooxygenase 1 and 2 (COX-1 and COX-2) are also essential for homeostasis and AA metabolism.^97^ COX-1 is involved in maintaining body homeostasis and protecting the gastric mucosa, whereas COX-2 is mainly involved in the inflammatory response.^98^

PAF-AH, also known as lpPLA_2_, is involved in the hydrolysis of the acyl ester in the sn-2 position of oxidized phospholipids, protecting cells from the toxic effects of these compounds.^99^ In the Lands cycle, ACSL activates fatty acids for reacylation, facilitating the reconstitution of membrane phospholipids.^100^ Together with Prdx6, an enzyme with antioxidant and calcium-independent phospholipase A2 activities, the integrity of membrane phospholipids is maintained, which is critical for maintaining proper cellular function.^101^

These enzymes and processes are interdependent and essential for lipid homeostasis and are also fundamental to liver and cardiovascular health. The balance between them is essential to prevent imbalances that lead to liver and cardiovascular disease and chronic inflammation. These enzymes are closely linked to the metabolism of polyunsaturated fatty acids such as AA, eicosapentaenoic acid (EPA), docosahexaenoic acid (DHA) and linoleic acid.^87^

Although AA is the preferred substrate for COX-1 and COX-2 enzymes, studies suggest that these enzymes can also metabolize other polyunsaturated fatty acids, albeit with less efficiency. This may occur particularly during oxidative stress, when the availability of these fatty acids in cell membranes increases. Other polyunsaturated fatty acids that can be metabolized by COX enzymes include EPA, DHA and linoleic acid.^102^

To better illustrate this cycle, we have developed Figure 3, which summarizes the complex interplay between fatty acid oxidation, membrane remodelling and inflammatory processes mediated by PAF and its receptors.

**Figure 3:**
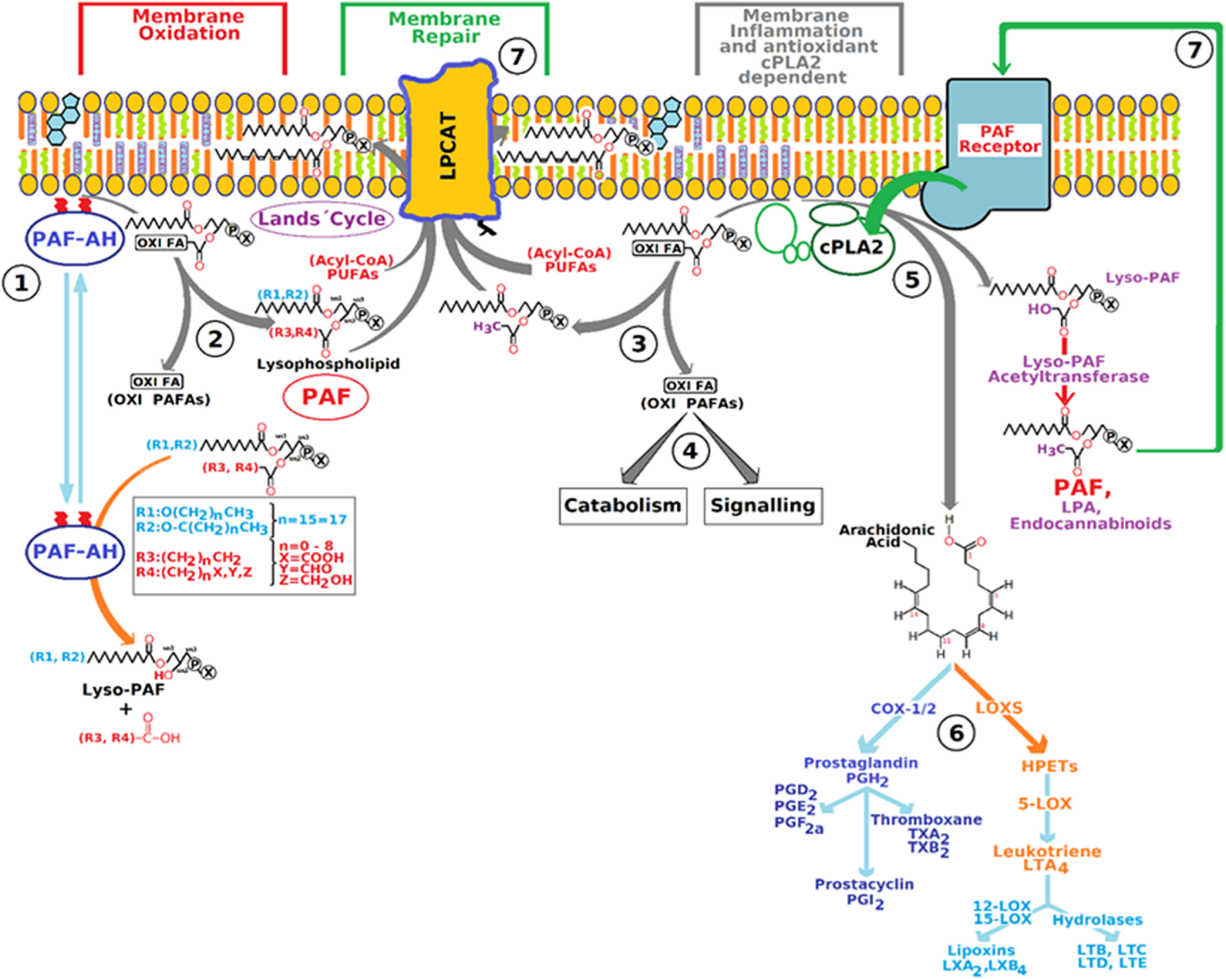
Stages and enzymes involved in the plasma membrane remodelling and repair process, focusing on phospholipids and inflammatory mediators.

The enzymes involved, such as lpPLA_2_, cPLA_2_, sPLA_2_ and LPCAT3, play critical roles both in maintaining membrane integrity and in mediating inflammatory responses, highlighting the importance of the Lands cycle in cellular homeostasis and in the response to cellular stress.

The PAF-AH action: Intracellular enzyme, similar to its extracellular isoforms, classified as a group VII PLA_2_, calcium independent and membrane associated. Its main function is to remove oxidized polyunsaturated fatty acids (OXY-FA) from membrane lipids, releasing PAF and oxidized fatty acids, which are recycled for membrane remodelling. After the action of PAF-AH, lysophospholipids are reacylated, reintroducing the oxidized fatty acids into the Lands cycle for membrane repair and remodelling. The released PAF can be used for membrane remodelling or neutralized by other enzymes. cPLA_2_ removes long-chain oxidized fatty acids, releasing PAF and oxidized fatty acids that can be remodelled or degraded. Oxidized fatty acids can be catabolized or involved in cell signaling, thereby amplifying inflammatory processes. PAF, released by the action of PAF-AH and cPLA_2_, can trigger the release of AA, which is metabolized to various inflammatory mediators such as prostaglandins and leukotrienes. AA is processed by enzymes such as COX and LOX (lipoxygenases) to produce inflammatory mediators important in acute inflammatory processes such as prostanoids, leukotrienes (LTs), epoxysatrienoic acids (EETs), dihydroxyeicosatrienoic acids (diHETEs), eicosatrienoic acids (ETEs) and lipoxins (LXs). LPCAT, an enzyme that reacylates lysophospholipids, is essential for maintaining the fluidity and functionality of cell membranes. Once synthesized, PAF can bind to the PAF receptor in the membrane and modulate various cellular responses, including inflammation.

The processes associated with the enzymes involved in this cycle play an essential role in maintaining the integrity of cell membranes and regulating inflammatory responses, with direct implications for lipid homeostasis and hepatic and cardiovascular health. The delicate balance between these enzymes and their activities is crucial for the prevention of chronic inflammation and the development of associated diseases, highlighting the importance of a thorough understanding of this cycle for the advancement of therapeutic strategies aimed at the prevention and treatment of inflammatory and metabolic disorders.

PLA_2_’s are enzymes that catalyze the hydrolysis of phospholipids, releasing fatty acids and lysophospholipids, with implications for inflammatory processes and membrane remodelling. In addition, acyltransferases such as LPCAT and medium/long-chain acyl-CoA synthetase (ACSM/ACSL) play critical roles in cellular lipid homeostasis and the Lands cycle. A detailed understanding of the different PLA_2_ isoforms and acyltransferases is essential for the development of new therapeutic approaches. The identification of specific inhibitors and the exploration of the clinical implications of these enzymes could lead to significant advances in the treatment of inflammatory, cardiovascular and cancer diseases.

## Phospholipase

PLA_2_ is a crucial enzyme in human lipid metabolism and plays a fundamental role in several physiological and pathological processes.^103^ This enzyme belongs to a superfamily of proteins that catalyze the hydrolysis of phospholipids at the sn-2 position, releasing fatty acids and lysophospholipids.^104^ In the human body, PLA_2_ is involved in several essential functions, including cell membrane remodelling, cell signaling and the production of bioactive lipid mediators. There are several isoforms of PLA_2_, each with different properties, such as sPLA_2_, cPLA_2_, LpPLA_2_ and calcium-independent PLA_2_ (iPLA_2_).^105^

PLA_2_ activity is particularly important in the production of AA, an essential precursor for the synthesis of eicosanoids such as prostaglandins and leukotrienes.^103^ These lipid mediators are essential for inflammatory processes, immune response and cellular homeostasis.^106^ In addition, PLA_2_ plays a critical role in maintaining the integrity of cell membranes, contributing to their fluidity and functionality.^104^

Studies have shown that alterations in PLA_2_ activity are associated with several pathological conditions, including inflammatory, cardiovascular, and neurodegenerative diseases.^105,107^ For example, overexpression or excessive activation of PLA_2_ has been associated with chronic inflammatory processes and tissue damage.^106^

Human PLA_2_ exists in several isoforms, each with specific characteristics in terms of cellular localization. Figure 4 shows the different structures of these isoforms that carry out regulation and physiological functions.^105^

**Figure 4:**
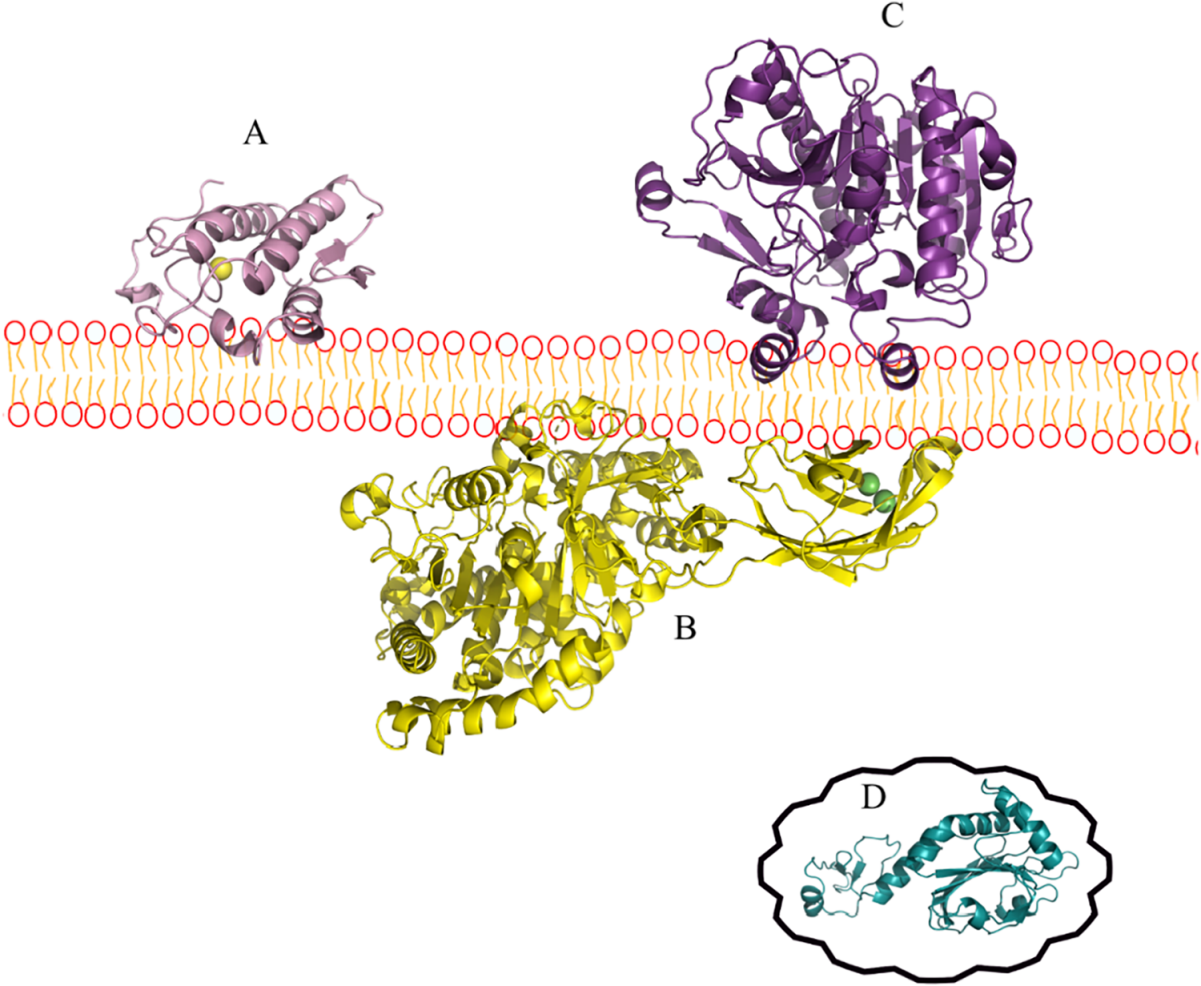
Three-dimensional structures of the Human PLA_2_ exists in several isoforms. (A) Secretory phospholipase A2 of *Homo sapiens*, represented in cartoon in purple and in green spheres represent Calcium. (B) Cytosolic phospholipase A2 of *H. sapiens*, represented in cartoon in yellow and in green spheres represent Calcium. (C) Platelet-activating factor acetyl hydrolase (Paf-Ah) of *H. sapiens*, represented in cartoon in dark purple. (D) Peroxiredoxin 6 of *H. sapiens*, represented in blue.

In addition to PLA_2_, phospholipase B (PLB) is also of great interest. PLB is a bifunctional enzyme that can remove both sn-1 and sn-2 fatty acids from phospholipids, resulting in glycerophosphorylcholine. It plays a key role in cellular processes involving membrane turnover and the response to cellular damage.^108^

In some infections, such as those caused by pathogenic fungi, PLB plays an important role in pathogen virulence by facilitating the penetration of the host membrane.^109^ Recent studies have explored modulation of PLA_2_ and PLB activity as a therapeutic strategy in inflammatory, cardiovascular and neurodegenerative diseases.^108,109^

The regulation of PLA_2_ activity is complex and involves multiple mechanisms, including phosphorylation, protein–protein interactions, and calcium ion modulation.^107^ This precise regulation is essential to maintain the balance between normal physiological functions and prevent the detrimental effects associated with excessive enzyme activity; alterations in the activity of these enzymes can disrupt membrane homeostasis, exacerbate inflammation and promote chronic disease.^106^

### Secretory Phospholipases A2

Secretory phospholipases A2 are a class of enzymes with critical functions in cell biology, particularly in the degradation of membrane phospholipids and in mediating inflammatory processes. These enzymes are small proteins, generally consisting of 120 to 150 amino acids, which may or may not be anchored to cell membranes. They have a single catalytic domain whose activity depends on calcium ions (Ca^2+^) and is highly specific for interaction with lipid substrates.^107^ The catalytic domain of sPLA_2_ contains a highly conserved catalytic triad consisting of Ca^2+^ ions, histidine (His) and aspartate (Asp) residues; a water molecule is required to stabilize each Ca^2+^ ion, which together form a ‘catalytic triad’. This configuration is essential for enzymatic catalysis, where Ca^2+^ acts as a cofactor, stabilizing the enzyme by binding to aspartate or glutamate residues to form a stable enzyme complex. Water plays a crucial role in the function of sPLA_2_, forming a hydration layer around calcium, which is necessary to stabilize the negative charge and maintain the correct conformation of the binding site.^108^

sPLA_2_ show a preferential affinity for membrane phospholipids, especially those rich in unsaturated fatty acids. This specific recognition is fundamental to their biological function, allowing the cleavage of the sn-2 ester of phospholipids—an essential step in the generation of free fatty acids, such as AA, and bioactive lysophospholipids, which play important roles in inflammatory processes and cell signaling.^108^

Interfacial activation, a phenomenon in which the catalytic activity of sPLA_2_ increases significantly in the presence of a lipid interface, is also critical for its biological function, facilitating the access and hydrolysis of membrane phospholipids.^10^ During the catalytic process, the Ca^2+^ ion, coordinated by aspartic and glutamic acid residues and water molecules, ensures the correct conformation of the binding site and stabilizes the enzyme complex.

In the transition state, calcium stabilizes the negative charge formed, while histidine in the active site facilitates the hydrolysis of the sn-2 ester. Residues such as Tyr52 and Tyr73 interact with His48, Asp49 and Asp99, directly influencing the dynamics of enzymatic catalysis, while additional residues such as Gly29, Gly30 and Lys60, located in the “calcium loop”, are critical for the maintenance of calcium in the enzyme and consequently for its catalytic activity.^110,111^

Inhibition studies also highlight the importance of these residues. The commercial inhibitor Varespladib (LY315920), an efficacy in inhibiting sPLA_2_ by binding to critical regions involved in calcium maintenance such as Asp49, Lys60, Gly29 and Gly30, resulting in a reduction in inflammatory events associated with the release of fatty acids and lysophospholipids. In addition to small molecule inhibitors, peptide mimetics are being developed to block the active site or alter the conformation of the enzyme, offering new opportunities for therapeutic intervention in inflammatory diseases.^111^

sPLA_2_ are enzymes of high biological relevance, playing a vital role in inflammatory processes and other pathologies. A detailed understanding of their structure, mechanisms of action and interaction with inhibitors opens new perspectives for the development of specific therapies. Interfacial activation, a phenomenon in which the catalytic activity of sPLA_2_ increases significantly in the presence of a lipid interface, is also critical for its biological function, facilitating the access and hydrolysis of membrane phospholipids.^10^

### Lipoprotein-Associated Phospholipase A2

Lipoprotein-associated phospholipase A2, also known as PAF-AH (platelet-activating factor-acetyl hydrolase), is an important enzyme in molecular biology and medicine, particularly in vascular inflammation and atherosclerosis. This evolutionarily conserved enzyme has a catalytic structure typical of α/β-hydrolases, with a catalytic triad consisting of serine, aspartic acid and histidine.^112^ PAF-AH has a catalytic triad composed of Ser273, His351 and Asp296, analogous to that found in serine proteases, where histidine facilitates the hydrolysis of acyl groups. The enzyme, with a molecular weight of 45–50 kDa, associates mainly with plasma lipoproteins such as LDL (low-density lipoprotein) through residues such as Trp115 and Leu116, and plays a crucial role as a biomarker in vascular inflammation and atherosclerosis.^91,112^

In addition to the catalytic triad, PAF-AH has a secondary pocket formed by these eight residues, such as Phe110, Leu153, Phe274, Trp298, His272, Tyr160, Gln352 and Phe322, which stabilize the substrate or regulate substrate binding, giving the enzyme a selective affinity for it. PAF-AH is known to covalently interact with the negative charge of the paraoxon byproduct diethyl phosphate (DEP). DEP is an organophosphate byproduct that covalently binds to serine in the active site, demonstrating the critical role of the enzyme in the degradation of toxic compounds and in the regulation of the inflammatory response.^105^

The secondary catalytic pocket surrounding the catalytic triad confers selective affinity to PAF-AH for specific substrates and is essential for enzymatic function in different environments. The binding of DEP to serine highlights the critical role of this enzyme in the degradation of potentially toxic compounds and in the regulation of the inflammatory response. The main catalytic function of PAF-AH is the hydrolysis of oxidized phospholipids and platelet activating factor (PAF). This process is mediated by the removal of the acetyl group at the sn-2 position of PAF, producing Lysol-PAF and acetate. This enzymatic activity is critical for the modulation of inflammation, as both PAF and oxidized phospholipids are potent inflammatory mediators.^105,113^ Therefore, we can observe on Figure 5 the comparison of interaction between the commercial inhibitor Darapladib and the covalent inhibitor DEP with the catalytic pocket of the enzyme.

**Figure 5:**
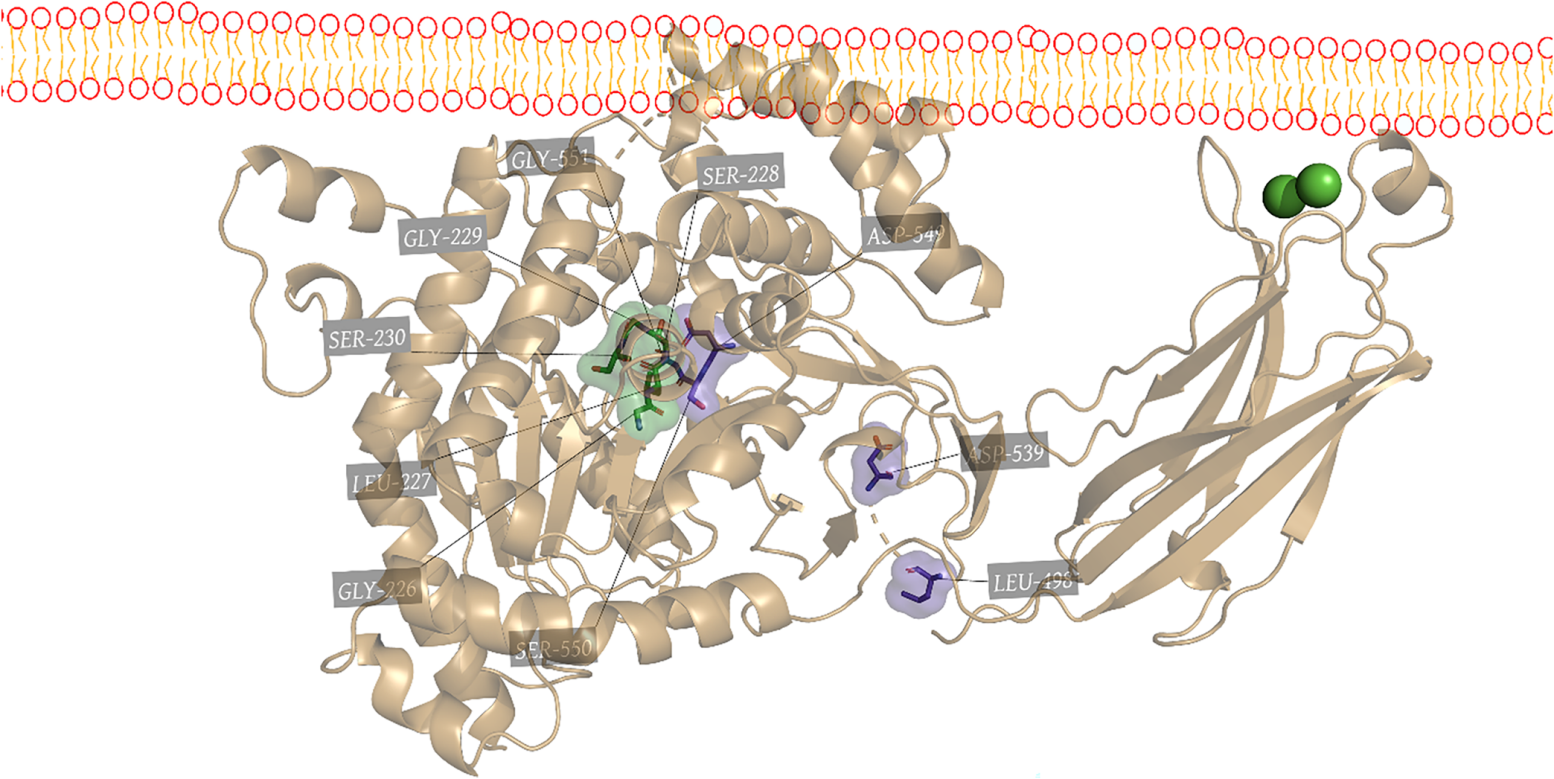
Human cytosolic PLA_2_ on the membrane the stickers representing a catalytic pocket in purple and green.

Three major PAF-AH isoforms have been identified: plasma PAF-AH, PAF-AH II and PAF-AH Ib. The plasma PAF-AH isoform is a monomeric protein with a molecular weight of 45 kDa, whereas PAF-AH II is an intracellular enzyme of 40 kDa, predominantly expressed in the liver and kidneys. PAF-AH II shares 41% sequence identity with plasma PAF-AH and both do not require Ca^2+^ for enzymatic activity^91^ Although the isoforms share the same active site (Ser273, His351 and Asp296), their functions differ depending on the cell type and the intracellular or extracellular environment. PAF-AH can act on several substrates in addition to PAF, including oxidized lipids, suggesting a functional versatility adapted to the specific physiological needs of different tissues.^112,114^

Darapladib is a commercially available inhibitor that blocks PAF-AH activity by interfering with both the catalytic triad and the secondary pocket. Due to its size and structure, Darapladib prevents the substrate from interacting with critical residues in the active site, including Phe110, Leu153 and Phe274. This inhibition results in effective suppression of PAF-AH enzymatic activity, with potential implications for the treatment of cardiovascular and inflammatory diseases.^115^ In addition to Darapladib, there is growing interest in the development of specific inhibitors that modulate the activity of different PAF-AH isoforms to treat diseases with exacerbated inflammatory components.

Understanding the structure and dynamics of the catalytic triad and the secondary pocket is fundamental to the rational design of new inhibitors with greater selectivity and reduced toxicity.^105,112–115^ Phospholipase A2 is essential for the control of inflammation and the prevention of atherosclerotic disease. Its mechanism of action, which is highly dependent on the catalytic triad and its interaction with lipoproteins, makes it an important therapeutic target. The study of its isoforms and the specific inhibition of its catalytic activity offer new perspectives for the treatment of various inflammatory and cardiovascular diseases.

### Cytosolic Phospholipase A2

Cytosolic phospholipase A2 (cPLA_2_) plays a key role in cellular processes, including inflammatory signaling, by releasing AA from membrane phospholipids. It is significantly larger and more complex than other phospholipase A2s, such as sPLA_2_, due to the presence of more than 700 residues and specialized domains that confer its functionality.^116^

It is an enzyme with a predicted molecular mass of 85.2 kDa, composed of two main domains: the C2 domain and the phospholipase catalytic domain. The C2 domain of approximately 130 residues is critical for calcium-dependent binding to the membrane, facilitating the interaction of the enzyme with its substrates. This domain has a β-sandwich structure consisting of 8 β-strands that coordinate two calcium ions. These ions bind to a specific cavity, allowing the enzyme to anchor to the membrane and ideally position the substrate for catalysis.^117,118^

The catalytic domain of cPLA_2_ is responsible for phospholipid hydrolysis and contains an essential catalytic triad formed by residues Ser727, His486 and Asp541. In some species, histidine (His486) can be replaced by arginine (Arg200), a functionally conserved substitution, as the guanidine group of arginine can play a catalytic role similar to that of histidine, maintaining the enzymatic activity and structural stability of cPLA_2_.^119^ The C2 domain of cPLA_2_ is responsible for its membrane binding, which is mediated by the presence of calcium. This domain positions the enzyme so that the substrate is correctly oriented for hydrolysis. Several amino acid residues in the C2 domain and along the membrane-binding interface are critical for this interaction, including Arg228, Lys221, Glu589, Asn555 and Phe671. These residues contribute to the stability of cPLA_2_ in the membrane by facilitating electrostatic and hydrophobic interactions that are essential for its function as we can see on Figure 5.^90,105,120^

The constructive interaction between residues at the membrane interface of cPLA_2_ is essential for its catalytic activity. For example, Arg228 interacts electrostatically with phosphatidylserine (PS) phosphates, while Lys221 stabilizes membrane binding. Residues such as Glu589 and Asn555 are critical for stabilizing the enzyme complex through hydrogen bonding, while hydrophobic residues such as Phe671 and Leu421 promote interactions necessary for maintaining the active conformation of the enzyme. This coordinated interaction ensures the catalytic efficiency of cPLA_2_ and its function in cell signaling.^121^

cPLA_2_ plays a crucial role in the release of AA, a precursor of eicosanoids such as prostaglandins and leukotrienes, which are important mediators in inflammatory processes. The activity of cPLA_2_ is therefore directly linked to the modulation of inflammatory responses and the regulation of cellular events associated with stress and inflammation.^77^ Given its vital role in inflammation, cPLA_2_ is a target enzyme in several pathological conditions, including cardiovascular, neurodegenerative and autoimmune diseases. Regulation or inhibition of cPLA_2_ can potentially attenuate exacerbated inflammatory responses, making it a focus of interest in the development of anti-inflammatory therapies.^123^

Several compounds have been identified as cPLA_2_ inhibitors, including girapladib and indomethacin heptyl ester (IHE), which act at the catalytic site to inhibit enzymatic activity.^116–118^ In addition, 2-(*N*-morpholino)-ethanesulfonic acid specifically inhibits the C2 domain, preventing cPLA_2_ from binding to the membrane. Selective inhibition of these domains holds promise for the development of treatments aimed at reducing the production of inflammatory mediators.^124^

The design of new cPLA_2_ inhibitors is focused on increasing specificity and reducing the side effects associated with enzyme inhibition. Molecular structures based on the interaction of the C2 domain with the membrane and the catalytic triad may offer new avenues for modulation of cPLA_2_ with potential applications in chronic and acute inflammatory diseases.^122,125^

As cPLA_2_ is a multifunctional enzyme essential for the release of AA and the regulation of inflammation, its complex structure with specialized domains allows precise interactions with cell membranes and specific substrates and is targeted by inhibitors with significant therapeutic potential. Continued research into cPLA_2_ may lead to the development of new anti-inflammatory therapies with applications in a wide range of diseases.

### Intracellular Calcium-Independent Phospholipase A2

Intracellular calcium-independent phospholipase A2 (aiPLA_2_), also known as peroxiredoxin 6 (Prdx6), is a multifunctional enzyme that plays a crucial role in several cellular processes. This enzyme, known for its activity as both a phospholipase and a peroxiredoxin, is predominantly localized in lysosomes, particularly in macrophages, where it operates under pH4 conditions. In addition to its phospholipase functions, Prdx6 also exhibits chaperone and antioxidant activities that are regulated by signaling pathways such as MAPK. These properties make Prdx6 a “moonlighting” enzyme, i.e., capable of performing several different biological functions.^125,126^

Prdx6 is a relatively small protein consisting of approximately 224 amino acids with an approximate molecular weight of 25 kDa. Its structure contains tryptophan residues (Trp33, Trp82 and Trp181) which provide the structural flexibility necessary for its multiple functions. Peroxiredoxin activity is mediated by key residues such as cysteine 47 (Cys47) and histidine 39 (His39), while phospholipase activity is catalyzed by a triad of histidine 26 (His26), aspartate 140 (Asp140) and serine 32 (Ser32), with cysteine 2 (Cys2) playing a critical role in both catalytic sites. In particular, Ser32 is highly conserved across species, and although it is replaced by cysteine in plants, it retains its essential function in phospholipid activity.^127,128^

Other amino acids play key roles in the structure and function of Prdx6. Residues such as arginine 22 (Arg22), isoleucine 23 (Ile23) and arginine 24 (Arg24) help to stabilize the structure and interact with substrates through electrostatic and hydrophobic interactions. Aspartate 27 (Asp27), together with Aspartate 104 (Asp104), helps to stabilize the active site and maintain the catalytic environment. Residues such as arginine 106 (Arg106) and asparagine 107 (Asn107) are important for substrate binding and stabilization of the enzymatic confirmation. Methionine 127 (Met127) and proline 128 (Pro128) confer structural rigidity and stabilize internal interactions of the protein, which is crucial for the integrity of the three-dimensional structure of Prdx6 and consequently for its biological functions.^81,82^

The structural flexibility of Prdx6, conferred by the tryptophan residues, is crucial for its moonlighting ability. This flexibility allows the enzyme to change conformation in response to changes in the cellular environment, such as pH or intracellular signaling, exposing different active sites and facilitating interactions with specific substrates. Ser32 is particularly important, for the phospholipid function of the enzyme, as it is essential for catalysis. It is often targeted by inhibitors such as the literature-recommended MJ33-OH and the ChemBL-recommended Pcbp2-gpx4, which suppress phospholipid activity without affecting other functions of Prdx6.^94,129^

Prdx6 plays a role in a variety of cellular environments, acting as an antioxidant by reducing lipid peroxides and as a repairer of membranes damaged by lipid peroxidation. During oncogenesis, Prdx6 becomes particularly active, facilitating the repair of cell membranes and promoting survival in adverse conditions such as the tumor environment. This function is critical in the metastatic stages, where cell membrane integrity is essential for cell invasion and migration.^129^

In oncogenesis, dysregulation of Prdx6 activity is associated with a variety of pathologies. Increased or inappropriate levels of Prdx6 activity have been implicated in tissue injury, sepsis, central nervous system disorders, diabetes and male infertility. In particular, its ability to repair membrane damage and reduce ROS makes it an enzyme of great interest in both cell protection and pathogenesis.^130^

Major substrates of Prdx6 include membrane phospholipids and lipid peroxides, which are essential for maintaining cellular homeostasis. An important ligand identified for Prdx6 is Pcbp2-gpx4, which plays a role in the regulation of lipid peroxidation. Regulation of Prdx6 activity is therefore critical for the control of lipid peroxidation and cell membrane integrity under normal and pathological conditions.^131^

Among the selective inhibitors of Prdx6 phospholipid activity, the compound MJ33 OH stands out. This inhibitor acts specifically on the phospholipid catalytic site of the enzyme, suppressing its activity without interfering with peroxiredoxin or chaperone functions. The ability to selectively inhibit different activities of Prdx6 provides a powerful tool for studying its biological functions and developing potential therapies for pathologies associated with its dysregulation.^131^

Intracellular calcium-independent phospholipase A2, or Prdx6, is a multifunctional enzyme essential for maintaining cellular integrity and responding to oxidative stress.

Its ability to act in different cellular environments, combined with its diverse enzymatic activities, makes it a critical enzyme in normal and pathological processes, including oncogenesis and other inflammatory and degenerative diseases.^128^

### Calcium-Independent Phospholipase A2 and Glutathione Peroxidase 4 AXIS

The interaction between Glutathione Peroxidase 4 (GPX4) and calcium-independent Phospholipase A2 (iPLA_2_-VIA) forms a critical regulatory axis for lipid homeostasis and cellular response to oxidative stress, with direct implications for ferroptosis.^132–134^ These enzymes act antagonistically yet complementarily to prevent the accumulation of oxidized lipids and ferroptotic cell death.^132,134–136^

GPX4 is a unique antioxidant enzyme from the glutathione peroxidase family, capable of directly reducing lipid hydroperoxides within cellular membranes, thereby halting the propagation of lipid peroxidation—a key driver of ferroptosis.^132,134,135^ GPX4 utilizes reduced glutathione (GSH) as a cofactor to catalyze the reduction of hydroperoxides (ROOH) to their corresponding alcohols (ROH), preserving the structural integrity of lipid bilayers and cell viability.^132,134^ Inhibition or deficiency of GPX4 leads to the irreversible accumulation of lipid peroxides such as 15-HpETE-PE, triggering ferroptosis, a form of regulated, iron-dependent cell death that is mechanistically distinct from apoptosis or necrosis and relevant to neurodegeneration, ischemia-reperfusion injury, and cancer microenvironments.^134,137^ GPX4 knockout is embryonically lethal in mouse models, emphasizing its essential function.^134,138^

Crystal structures of GPX4 (PDB: 5H5Q,^8^ 6HKQ,^139^ 7U4N,^140^ 8Q8J^141^) reveal a globular protein with a hydrophobic ring that anchors the enzyme to peroxidized membranes, facilitating efficient catalysis.^78,132,142^ Residue Leu130 inserts into the lipid bilayer, optimally positioning the active site near substrates. The catalytic triad comprises Sec46, Gln81, and Trp136, where selenocysteine (Sec46) acts as a redox-active center. This residue is more reactive than cysteine due to the lower ionization energy of selenium. Upon oxidation by lipid hydroperoxides, Sec46 forms a selenenic acid (SeOH), which is then reduced back to its active form by two equivalents of GSH, forming oxidized glutathione (GSSG).^134,137,143^

While GPX4 detoxifies lipid peroxides, iPLA_2_-VIA cleaves damaged fatty acids for re-esterification, thus creating a protective membrane repair axis.^133,144,145^ Under stress conditions, such as GPX4 inhibition or iron overload, this interplay may paradoxically result in the release of oxidizable free PUFAs, promoting ferroptosis.^132,136,137,146^

iPLA_2_-VIA functions as a lipid repair enzyme, selectively removing oxidized fatty acids from membrane phospholipids, particularly from the sn-2 position of glycerophospholipids. This activity is calcium-independent and works alongside re-esterification enzymes such as LPCATs to replenish damaged lipids with functional species.^135,147^ Although protective under physiological conditions, iPLA_2_-VIA activity can also liberate free PUFAs like arachidonic acid, which may serve as substrates for peroxidation under ROS or lipoxygenase activity, thereby sensitizing cells to ferroptosis.^68,82^

Despite limited structural data for human iPLA_2_-VIA, homologous models and other PLA_2_ enzymes, such as bee venom PLA_2_ (PDB: 1POC),^148^ illustrate a catalytic His/Asp dyad and lipid-interfacial binding domains.^73,149^ iPLA_2_-VIA is thought to contain a flexible catalytic pocket with a Ser–His–Asp triad suited for oxidized phospholipids. It also includes ankyrin repeat motifs that mediate membrane association, redox-sensitive phosphorylation sites, and other regulatory domains, linking its activity to the cellular redox environment.^133,134,150^

Unlike calcium-dependent PLA_2_s, iPLA_2_-VIA exhibits conformational flexibility to accommodate membrane damage, preferentially acting on peroxidized phospholipids.^147^ The functional relationship between GPX4 and iPLA_2_-VIA is highly context-dependent, governed by the redox balance and lipid damage burden. Simultaneous inhibition of both enzymes markedly increases cellular susceptibility to ferroptosis, a strategy currently under investigation for targeting apoptosis-resistant tumors.^68,146,149^

Additionally, redox-modulating drugs like aspirin and natural compounds can alter GPX4 and iPLA_2_-VIA expression and activity, offering therapeutic avenues for ferroptosis modulation in inflammatory diseases and cancer.^151–154^ Pharmacological inhibition or genetic knockout of GPX4 consistently results in lipid peroxide accumulation and ferroptosis cell death.^152,155,155^

The GPX4/iPLA_2_-VIA axis represents a dynamic homeostatic mechanism: GPX4 provides frontline protection against lipid peroxidation, while iPLA_2_-VIA manages residual lipid damage.^78,133,156^ Imbalance in this axis predisposes cells to ferroptosis, which is emerging as a therapeutic target in diseases like Parkinson’s and cancer.^152,154,157^ Continuous activity of iPLA_2_-VIA may also contribute to free PUFA buildup, which is especially prone to oxidation in the presence of ferric iron (Fe3+), amplifying oxidative injury.^78,134,158^

Combined strategies targeting GPX4 and iPLA_2_-VIA may be effective in inducing ferroptosis in tumor cells, especially those resistant to apoptosis. Conversely, enhancing their activity might protect nervous or pulmonary tissues from oxidative damage. Understanding this enzymatic axis is critical for developing targeted therapies against ferroptosis-mediated pathologies, including cancer, neurodegeneration, and ischemia-reperfusion injury.^78,159^

## Acyltransferases

Acyltransferases are a class of enzymes that play a fundamental role in cell biology by facilitating the transfer of acyl groups, often derived from fatty acids to various acceptor molecules. This process is essential in many metabolic pathways, including lipid biosynthesis, post-translational modification of proteins and regulation of cell signaling. Due to their broad relevance, acyltransferases are an important focus of study in biochemistry and cell biology.

These enzymes exhibit a wide structural diversity, influenced by their cellular localization and specific function. However, one aspect they have in common is the presence of a highly conserved active site, which is essential for recognition and interaction with their specific substrates, facilitating the catalysis of acyl group transfer. In many acyltransferases, the active site contains amino acid residues that play a critical role in the activation of the acyl group and the stabilization of reaction intermediates.^100,160^

The mechanism of action of acyltransferases generally follows a pattern of reactions involving the formation of a tetrahedral intermediate. First, the acyl group at the active site of the enzyme is activated, often by the formation of a thioester or intermediates, depending on the type of acyltransferase. This activated intermediate facilitates the transfer of the acyl group to a functional group on an acceptor molecule, such as a hydroxyl or amine group. The reaction is completed with the release of the acylated product, which may be a lipid, an acylated protein or another modified compound.^161^

The formation of the tetrahedral intermediate is a critical step in the acylation mechanism. During this process, the interaction between the acyl group and the substrate occurs at the active site, where specific amino acid side chains stabilize the transition state and facilitate the reaction. This mechanism is often compared to that of serine proteases, where an acyl-enzyme intermediate is formed, highlighting the importance of serine, cysteine or histidine residues in the active site.^162^

After the formation of the tetrahedral intermediate, the enzyme cleaves the intermediate, resulting in the release of the acylated product. This product is then released from the enzyme, completing the catalytic cycle. The efficiency of this step is crucial for the overall activity of the acyltransferase and can be modulated by factors such as pH, the presence of cofactors and protein–substrate interactions.^163^

Acyltransferases exist in both soluble and membrane-associated forms. The cellular localization of these enzymes is closely related to their biological function. For example, membrane-associated acyltransferases play a key role in the biosynthesis of phospholipids and other cell membrane components. In contrast, soluble acyltransferases may be involved in the modification of cytosolic proteins or in the regulation of intracellular signaling. The subcellular distribution of these enzymes reflects the diversity of their roles in the cell, ranging from the synthesis of lipid complexes to the modulation of protein function.^164^

These enzymes are often involved in the biosynthesis of membrane lipids, including phosphoglycerates and sphingolipids. Membrane association allows these enzymes to interact directly with their lipid substrates, facilitating the insertion of new lipids into the cell membrane and maintaining its structural integrity.^165^

Soluble acyltransferases play a critical role in the post-translational modification of proteins, such as the addition of acyl groups that affect the subcellular localization and activity of proteins. In addition, these enzymes can be involved in the regulation of cellular signaling pathways, acting as modulators of protein function by acylating specific residues of signaling proteins.^166^

Acyltransferases play an essential role in cellular homeostasis and adaptation to the environment. Dysfunction of these enzymes can lead to a variety of diseases, including metabolic disorders, neurodegenerative diseases and cancer.^1,130^

Because of their leading role in lipid biosynthesis and protein modification, acyltransferases are potential targets for pharmacological therapies. Specific inhibitors or modulators of these enzymes are being investigated as potential treatments for a variety of diseases, including type 2 diabetes, dyslipidaemia and certain cancers. A detailed understanding of the mechanism of action of these enzymes is therefore essential for the development of effective therapeutic strategies.^161,164^

Acyltransferases are versatile enzymes with critical functions in various metabolic pathways and cellular processes. Their ability to mediate the transfer of acyl groups is central to the maintenance of cellular integrity and the modulation of protein activity and cell signaling.

### Lysophosphatidylcholine Acyltransferases

#### Lysophosphatidylcholine Acyltransferase 1

LPCAT1 is an enzyme belonging to the acyltransferase family, which is involved in the Lands cycle. The Lands cycle is a dynamic mechanism of phospholipid remodelling that is essential for maintaining the structural and functional integrity of cell membranes.^1,167,168^

Its activity plays a pivotal role in maintaining the physicochemical properties of the lipid bilayer, in addition to contributing to the adaptive response to oxidative stress. LPCAT1 has been demonstrated to play an active role in the reincorporation of oxidized phospholipids, thereby facilitating the restoration of membrane fluidity and asymmetry, in addition to modulating functional domains, including lipid rafts and the lateral distribution of transmembrane proteins.^65,169^

Its primary location is in the cytosol and endoplasmic reticulum, with elevated expression in epithelial tissues, particularly in type II pneumocytes, where it plays a crucial role in the biosynthesis of pulmonary surfactant.^170^ In contrast to LPCAT3, which is predominantly expressed in metabolic tissues such as the liver and the intestine, LPCAT1 exhibits functional specificity in the generation of saturated PCs (e.g., 16:0/16:0), which are essential for the mechanical stability of membranes in environments exposed to oxidative stress, such as lung tissue.^171^

The LPCAT1 gene, which is located on the short arm of chromosome 5 (5p15.2), is responsible for encoding LPCAT1,^172^ while the LPCAT3 gene, which is located on chromosome 12 (12q13.3), is responsible for encoding LPCAT3.^173^ The LPCAT1 gene is preferentially expressed in epithelial tissues and secretory cells, such as type II pneumocytes, while LPCAT3 is widely expressed in the liver, small intestine, and other tissues with high metabolic demand for lipids.^168,174,175^

Epigenetic alterations, somatic mutations, and genomic amplifications involving these loci have been associated with dysregulations in inflammatory and neoplastic processes. The differential activation of these genes contributes to cellular adaptation to oxidative or hypoxic environments, being regulated by pathways such as Nrf2, SREBP-1, and HIF-1α, which reinforces their role in the interface between lipid metabolism, cellular stress, and cell fate.^176,177^

LPCAT1 expression is subject to regulation by multiple transcription factors, including Nrf2, a master sensor of the antioxidant response, in addition to potential regulation by HIF-1α^178^ and c-Myc^179^ in hypoxic and metabolically reprogrammed tumor microenvironments.^180,181^

Post-transcriptional mechanisms have also been demonstrated to be involved, with microRNAs such as miR-378 and miR-455 playing a regulatory role in cancer and inflammation contexts.^182^

In conditions of oxidative stress, phospholipids containing PUFAs exhibit heightened susceptibility to peroxidation, resulting in compromised membrane integrity of the plasma and mitochondria. This process facilitates the induction of ferroptosis, a form of regulated cell death that is dependent on iron and lipid peroxides.^183^

In this context, LPCAT1 and LPCAT3 have been shown to contribute to the re-esterification of oxidized lysophospholipids, thereby promoting the renewal of lipid composition and mitigating the propagation of damage.^26^

However, LPCAT1 activity has also been shown to increase sensitivity to ferroptosis by incorporating PUFAs such as 20:4 (AA) and 22:6 (DHA) into phosphatidylcholine, generating substrates highly prone to peroxidation.^16^ The dual role of the enzyme is contingent upon the cellular context. In cells exhibiting reduced GPX4 activity or free iron accumulation, LPCAT1 has the capacity to amplify oxidative damage. Conversely, under physiological conditions, its function is predominantly protective.^27,184^

Transcriptomic and proteomic studies have revealed that LPCAT1 is frequently overexpressed in several types of cancer, including lung adenocarcinoma, hepatocarcinoma, glioblastoma and colorectal cancer.^29,59^ This expression is associated with classic tumor characteristics, such as increased cell proliferation, evasion of apoptosis, metabolic reprogramming, and resistance to oxidative stress. Further evidence indicates that LPCAT1 may exert its influence over chromatin organization through interactions with nuclear phospholipids, thereby affecting the expression of oncogenic and pro-inflammatory genes.^185^

In this scenario, LPCAT1 has been proposed as an emerging therapeutic target, mainly in strategies that aim to induce ferroptosis through the inhibition of GPX4 or the induction of iron overload.^186^ Conversely, in models of pulmonary and degenerative diseases, such as chronic obstructive pulmonary disease (COPD) and acute respiratory distress syndrome, LPCAT1 has been shown to exert protective effects, promoting epithelial regeneration and the maintenance of alveolar surfactant.^170^

Consequently, LPCAT1 fulfils a multifaceted role at the interface between lipid metabolism, oxidative stress, and the regulation of cell death. Its activity may be beneficial in normal tissues exposed to damaging agents, but it may also act as a permissive factor for ferroptosis in tumor or inflammatory cells. It is imperative to comprehend this functional equilibrium to facilitate the development of targeted therapeutic interventions. This is particularly salient in the context of selective induction of ferroptosis in resistant neoplasms and the protection of normal tissues against oxidative injury.^167,187,188^

Furthermore, several tumors exhibit simultaneous expression of LPCAT1 and LPCAT3, although with distinct pathophysiological functions, since LPCAT1 is mainly associated with proliferation and evasion of apoptosis, while LPCAT3 is more involved in modulating tumor lipid metabolism and sensitivity to ferroptosis. LPCAT3 also plays important roles in hepatic homeostasis, cholesterol regulation and energy metabolism, and is implicated in conditions such as type 2 diabetes. In contrast, LPCAT1 contributes to the control of epithelial membrane fluidity.^174,189,190^

Alterations in the functional balance between LPCAT1 and LPCAT3 have the potential to dramatically modulate cellular susceptibility to ferroptosis, representing a promising axis for pharmacological intervention. While increased LPCAT3 expression may sensitize resistant tumors, modulation of LPCAT1 may protect normal tissues from oxidative injury.^170,190,191^

#### Lysophosphatidylcholine Acyltransferase 3

The LPCAT3 is a transmembrane enzyme critical for phospholipid remodelling, particularly the reacylation of LPC to PC. Its activity is essential for the biosynthesis of membrane phospholipids, thereby influencing the lipid composition of cell membranes and cellular adaptation to environmental changes. This review discusses the structure, mechanism of action, tissue localization and biological and pathological implications of LPCAT3.^192^

LPCAT3 is a 52 kDa enzyme mainly localized in hepatocytes, enterocytes, adipocytes and lung cells. It is a transmembrane protein involved in the biosynthesis of phospholipids in cell membranes. Although LPCAT3 forms dimers, it is monomeric, suggesting that its catalytic functions are independent. Its structure contains three main functional sites: the hydrophobic site, the catalytically active site and the hydrophilic site, which work together to capture and insert phospholipids into the membrane.^193^

The catalytic mechanism of LPCAT3 involves the transfer of the acyl group from acyl-CoA to LPC, resulting in the formation of PC. The process begins with the binding of LPC to the active site of LPCAT3, where the free hydroxyl group at the sn-2 position of LPC is reacylated, forming PC again. This process is essential for maintaining the lipid composition of the membrane, which is critical for the stability and functionality of cells.^194^

The residues essential for the function of LPCAT3, Figure 6, are located in the active site of the enzyme. In particular, residues Asn352 and His388 play a critical role in catalytic activation. In addition, a tunnel formed by tyrosine residues (Tyr143, Tyr151, Tyr298 and Tyr394) stabilizes the fatty acid during the reaction. These residues are critical for catalysis, facilitating the precise interaction between the substrate and the enzyme through hydrophobic and electrostatic interactions.^192,193^

**Figure 6:**
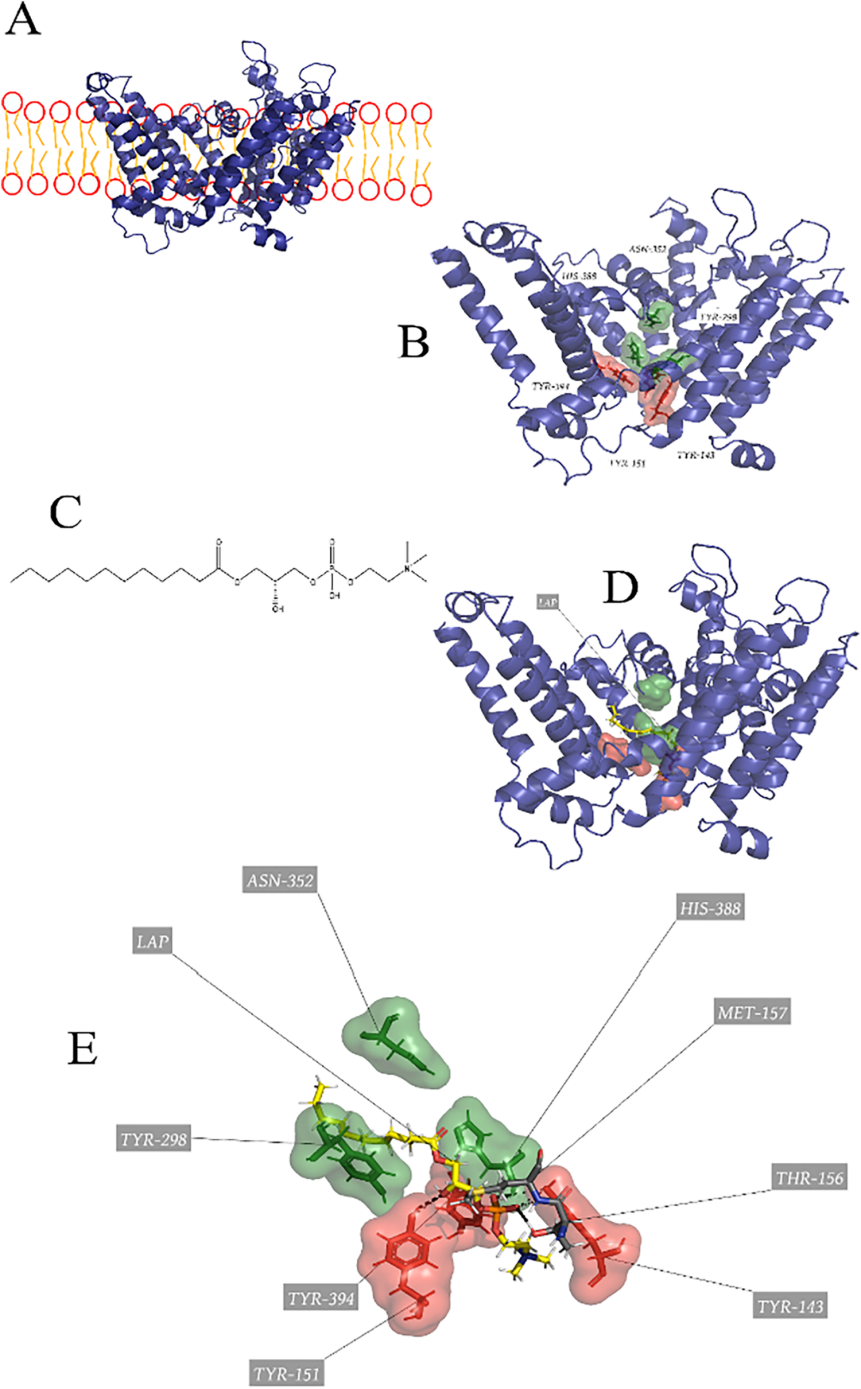
Human LPCAT3 with reference ligand. (A) Human LPCAT3, represented in the cartoon in blue inside membrane. (B) Human LPCAT3, the structure in green and blue represent the catalytic pocket in the triad catalytic and the secondary site in purple. (C) structure of the reference ligand LPC. (D) interaction between LPC on the catalytic site, represented in stickers in yellow the reference ligand structure, green the triad catalytic and purple the secondary site. (E) Pocket with the interactions on the catalytic site.

LPCAT3 plays a vital role in several tissues, including the liver, small intestine, skeletal muscle, macrophages and adipocytes. It is particularly important in the production of lipoproteins in the liver and small intestine, directly influencing lipid metabolism. Deficiencies in LPCAT3 activity in macrophages have been associated with increased production of inflammatory and atherogenic cytokines, suggesting a role in the pathogenesis of atherosclerosis.^194^

LPCAT3 dysregulation is associated with several pathologies, including cancer, diabetes and hyperlipidaemia. In colorectal cancer, LPCAT3 is used as a biomarker due to its high expression in plasma. Furthermore, in hyperlipidaemia, LPCAT3 dysregulation leads to excessive release of lipids into plasma, thereby increasing circulating lipid levels. In contrast, its inappropriate function may contribute to the development of diabetes, where lipid remodelling and cell membrane homeostasis are impaired.^195^

The role of LPCAT3 in cancer is of particular interest, especially in colorectal cancer, where its high expression is associated with tumor progression and metastasis. The enzyme contributes to cell membrane remodelling, which promotes cell survival and proliferation in the tumor environment. This remodelling is critical for cancer cell adaptation to changes in the microenvironment, facilitating invasion and metastasis.^196^

Although LPCAT3 inhibitors are still poorly understood, studies suggest that interference with its activity could affect phospholipid homeostasis, offering potential therapeutic targets for metabolic diseases and cancer. The use of RNA interference (RNAi) and other pharmacological agents to modulate LPCAT3 activity in preclinical models has shown promising effects in reducing plasma lipid burden and cancer progression.^193^

However, the lack of a detailed understanding of the mechanisms of inhibition limits the development of therapies based on LPCAT3 inhibitors. As its dysregulation is associated with several diseases, this highlights its importance as a potential therapeutic target. Further study of this enzyme will provide valuable insights into the development of pharmacological interventions for lipid-related diseases and cancer.

### Medium and Long-Chain Acyl-CoA Synthetase

Medium and long-chain acyl-CoA synthetases (ACSM/ACSL) are a class of enzymes essential for the activation of fatty acids by the formation of acyl-CoA. These enzymes play a crucial role in several metabolic pathways, including the Krebs cycle, β-oxidation and lipid biosynthesis. The formation of acyl-CoA is a necessary step for the utilization of fatty acids as an energy substrate and for the modification of lipid proteins.^197,198^

ACS are present in several cellular organelles, such as the mitochondrial outer membrane, peroxisomes and the endoplasmic reticulum, reflecting their involvement in multiple metabolic pathways. The structure of these enzymes includes a larger N-terminal domain of 517 residues and a smaller C-terminal domain of 130 residues, giving a total of 647 residues. This structural arrangement is essential for interaction with ATP and fatty acids, facilitating their activation.^199^

The fatty acid activation process begins with the binding of the fatty acid to an ATP molecule. The carboxyl oxygen of the fatty acid attacks the α-phosphorus of ATP, resulting in the formation of acyl-AMP and pyrophosphate, which is rapidly hydrolyzed into two inorganic phosphates. Next, the CoA group (CoASH) attacks the carbonyl of acyl-AMP, releasing AMP and forming acyl-CoA. This acyl-CoA is a key intermediate in several metabolic pathways, such as β-oxidation and lipid biosynthesis.^48,199^

The catalytic sites of ACS enzymes contain specific domains for the binding of ATP and fatty acid, which are essential for the activation of the fatty acid and the subsequent formation of acyl-CoA. Residues critical for this activity include Gly223, which contributes to the conformation of the active site; Lys555, which is involved in the interaction with ATP and is essential for the transfer of the phosphoryl group; Glu472 and Glu365, which stabilize the acyl adenylate intermediate; Tyr361, which facilitates the binding of the fatty acid to the active site; and Asp576, which is involved in the interaction with ATP and fatty acids and stabilizes the intermediate complex. These residues are essential for catalysis, we can see they interact with CoA on the Figure 7, interacting with ATP and fatty acids to facilitate the formation of the acyl adenylate intermediate, which is crucial for the subsequent transfer of the acyl group to CoA.^88,162,199^

**Figure 7:**
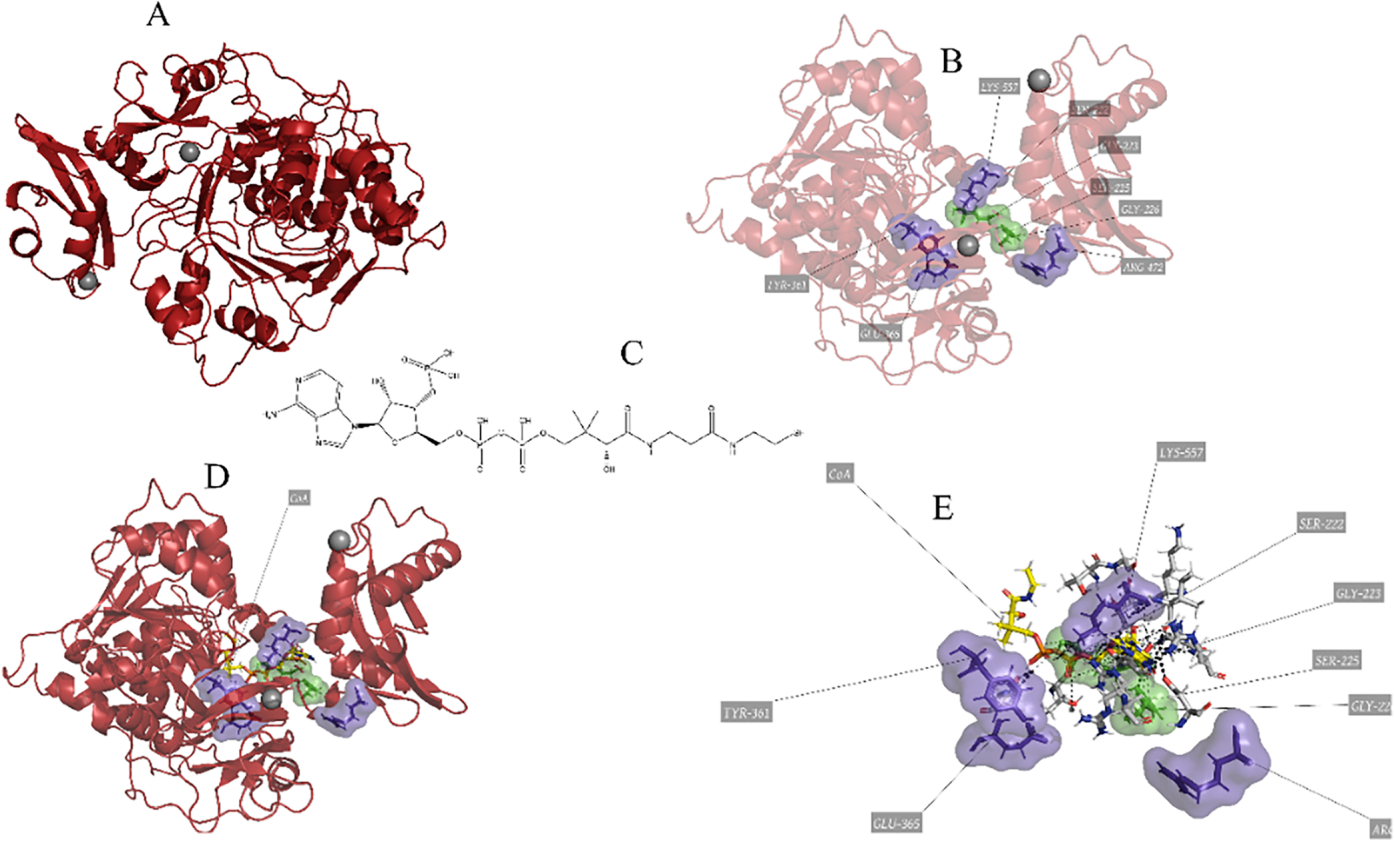
Human ACSMs and the structure of COA, a reference ligand that interacts directly in the catalytic pocket. (A) Human ACSMs in red with the Magnesium in gray and the structure of COA. (B) Human ACSM with the catalytic site, the structure in green, and blue represent the catalytic pocket in the triad catalytic and the secondary site in purple. (C) Structure of the reference ligand CoA. (D) Interaction between CoA on the catalytic site, represented in stickers in yellow the ligand structure, green the triad catalytic and purple the secondary site. (E) Pocket with the interactions on the catalytic site.

They show specificity with respect to the length of the fatty acid chain, which is reflected in the nomenclature that distinguishes these isoforms. Although the substrate specificity may vary, the basic catalytic function remains the same: to stroke medium to long chains to form acyl-CoA. This specificity influences the metabolic pathways in which each isoform is predominantly involved, with ACSMs preferring medium-chain fatty acids and ACSLs preferring long-chain fatty acids.^48^

They exhibit specificity with respect to the chain length of fatty acids, which is reflected in the nomenclature that distinguishes these isoforms. Although the substrate specificity may vary, the basic catalytic function remains the same: medium to long chain stroke to form acyl-CoA. This specificity influences the metabolic pathways in which each isoform is predominantly involved, with ACSMs preferring medium-chain fatty acids and ACSLs preferring long-chain fatty acids.^39^

Potential inhibitors include fatty acid analogues or molecules that interfere with the binding of ATP to the active site of the enzyme.

These inhibitors block the activation of fatty acids, thereby interfering with potentially critical metabolic processes, and therefore represent therapeutic targets in diseases related to lipid metabolism.^92,200,201^

Medium- and long-chain acyl-CoA synthesis is therefore an essential enzyme for the activation of free fatty acids, directly influencing several important metabolic pathways. Understanding its structure, mechanism of action and substrate specificity provides excellent insights into its role in cellular metabolism and its potential implications in metabolic diseases.

## Oxidative Stress, Inflammation, and Lipid Remodelling

Oxidative stress is linked to increased production of ROS and is widely recognized as a key event in the development of aging and several inflammation-related chronic diseases. When ROS production exceeds the total antioxidant capacity of the cell, one of the most immediate consequences is the oxidation of lipids-particularly polyunsaturated fatty acids, which are abundant in cell membranes. These structures become prime targets for oxidative damage, triggering chain reactions such as lipid peroxidation.^195,202^

During this process, ROS reacts with membrane phospholipids, leading to the formation of lipid hydroperoxides and alkyl radicals. These changes affect the fluidity and structural integrity of the membrane, leading to cellular dysfunction.^203^

In this context, the Lands cycle—the mechanism responsible for the continuous remodelling of membrane phospholipids—plays a crucial role. The enzyme cPLA_2_ removes PUFAs from the sn-2 position of phospholipids, forming lysophosphatidylcholine (LPC) and AA. This enzyme is particularly sensitive to oxidative signals and is activated by oxidative stress, which stimulates the production of AA, platelet-activating factor (PAF) and consequently pro-inflammatory eicosanoids.^195,204^

Oxidative stress and lipid remodelling form a dynamic network that influences both physiological and pathological processes. During inflammation, the Lands cycle regulates the lipid composition of cell membranes by modulating the activation of receptors such as toll-like receptors. The action of cPLA_2_ releases arachidonic acid, which is converted into pro-inflammatory mediators such as prostaglandins and leukotrienes. These mechanisms are linked by the Lands cycle, a central system for the renewal of membrane phospholipids with implications ranging from cell signaling to the development of chronic disease. This cycle acts in synergy with the Kennedy pathway, which is responsible for the de novo synthesis of phosphatidylcholine, which is subsequently remodelled by the Lands cycle to adjust the composition of fatty acids.^165^

PAF (1-*O*-alkyl-2-acetyl-*sn*-glycero-3-phosphocholine) is a bioactive phospholipid produced by various immune cells—including mast cells, basophils, eosinophils, macrophages and lymphocytes—as well as platelets and endothelial cells. By binding on its specific G-protein-coupled receptor (PAFR), which is widely expressed on cells of the immune system, PAF mediates acute and chronic inflammatory processes. Its degradation is tightly regulated by the enzyme PAF-AH, which prevents excessive accumulation of PAF and limits inflammatory damage. Dysregulation of this mechanism is associated with diseases such as psoriasis, sepsis, asthma, anaphylaxis and cardiovascular disease.^205,206^

PAF synthesis occurs via two distinct pathways: the remodelling pathway, initiated by cPLA_2_ and enhanced during inflammation, and the de novo synthesis pathway, which is less active under basal conditions.^206,207^ In the remodelling pathway, cPLA_2_ acts on phosphatidylcholine to generate LPC and AA. LPC is then acetylated by LPCAT to form PAF. Two isoforms of LPCAT are involved in this process: LPCAT1 (constitutively expressed) and LPCAT2 (inducible), the latter being essential in inflammatory responses.^208^

The enzymatic cycle involving cPLA_2_ and LPCATs is sensitive to inflammatory signaling and increased intracellular calcium. These factors promote the translocation of cPLA_2_α to intracellular membranes such as the endoplasmic reticulum and nuclear membrane. This translocation depends on the interaction between the C2 domain of the enzyme and specific phospholipids, such as phosphatidylcholine. In addition, its enzymatic activation is enhanced by phosphorylation at specific residues (such as Ser505) mediated by kinases such as MAPKs and p38.^209,210^

These modifications increase both the release of AA and the generation of pro-inflammatory eicosanoids. In parallel, PAF is selectively degraded by enzymes of the PAF acetyl hydrolase family, in particular the plasma isoform PLA_2_G7 and the intracellular hepatic form PAFAH-II. These enzymes belong to the class of lipase/esterases and share a conserved α/β-hydrolase structure with a catalytic triad of serine, aspartic (or glutamic) acid and histidine. This structure allows the preferential recognition and efficient degradation of lipids with short acyl chains at the sn-2 position, such as PAF.^114,211^

The coordinated activity between cPLA_2_, LPCATs and PAF-AHs not only regulates the levels of inflammatory lipid mediators—such as PAF and AA—but also helps to maintain the fluidity and integrity of cell membranes. Although these molecules amplify inflammatory responses necessary for the body’s defense, their deregulation can exacerbate pathological conditions. Alterations in the Lands cycle have also been linked to the resistance of tumor cells to apoptosis, favoring both tumor progression and resistance to treatment.

The integration of oxidative stress, inflammation and lipid remodelling via the Lands cycle offers new therapeutic perspectives. A deeper understanding of these mechanisms may lead to the development of more precise approaches to the treatment of neurodegenerative diseases, cancer and metabolic disorders. This highlights the importance of multidisciplinary strategies that combine lipid biochemistry with translational medicine.

## Crosstalk with Other Metabolic Pathways

Signaling pathways associated with oxidative stress, inflammation and lipid remodelling interact closely with key metabolic processes, including glucose, amino acid, and nucleotide metabolism. This crosstalk is mediated by signaling molecules such as cytokines, eicosanoids, and redox mediators that modulate gene expression, cell cycle progression, and apoptosis. In addition, there is a remarkable interdependence between lipid metabolism and mitochondrial bioenergetics, directly influencing ROS production and cellular redox homeostasis. Disruption of this metabolic interplay contributes to the pathophysiology of disorders such as type 2 diabetes, obesity, and metabolic syndrome.^203^

Cellular lipid metabolism comprises a highly interconnected network of tightly regulated biochemical pathways, including the Lands cycle, the Kennedy pathway, sphingolipid synthesis, fatty acid oxidation, and lipophagy. Rather than operating independently, these pathways work in a coordinated manner to maintain lipid homeostasis, preserve membrane integrity and ensure adequate energy supply. They are also actively involved in signaling events and cellular adaptation mechanisms.^212,213^ The Lands cycle—also known as the phosphatidylcholine remodelling pathway—plays a central role in modifying the fatty acid composition of phospholipids, particularly phosphatidylcholine, a major component of cell membranes.^214^

This remodelling process influences membrane fluidity, affects the function of membrane-associated proteins and allows dynamic cellular responses to environmental stimulus. In parallel, the Kennedy pathway is responsible for the de novo synthesis of phospholipids, providing essential building blocks for membrane expansion and renewal.

Sphingolipid synthesis is another central pathway, generating bioactive lipids such as ceramide and sphingosine-1-phosphate, which play structural and signaling roles in cell proliferation, differentiation and apoptosis.^215^

Fatty acid oxidation, predominantly mitochondrial, provides both ATP and regulatory metabolites that influence gene expression and overall cellular metabolism.^216^ Complementing these pathways, lipophagy, a selective form of autophagy that targets lipid droplets, facilitates the release of fatty acids and the degradation of oxidized or damaged lipid species.^217^

The harmonious integration of these metabolic circuits allows cells to modulate lipid storage, maintain membrane composition, and respond effectively to metabolic challenges.^218^ For example, under conditions of increased energy demand, fatty acid oxidation is regulated and lipophagy is activated to mobilize energy substrates. At the same time, the Lands cycle fine-tunes phospholipid composition, while the Kennedy pathway ensures continuous phospholipid biosynthesis to support membrane turnover.^219,220^

These processes are also intricately linked to one-carbon metabolism - specifically the methionine and folate cycles which are critical for the methylation of phosphatidylethanolamine and the synthesis of phosphatidylcholine. This highlights the deep integration of lipid metabolism with core metabolic networks.^221^ Dysregulation of this balance contributes to cellular dysfunction and disease. In pathological conditions such as obesity, nonalcoholic fatty liver disease and lipodystrophies, dysregulation of lipid synthesis and oxidation promotes triglyceride accumulation, chronic inflammation, and cellular stress.^222,223^

Overactivation of the Kennedy pathway may drive excessive synthesis of neutral lipids, while fatty acid oxidation may be impaired. In addition, lipophagy may be insufficient to remove damaged lipid droplets, and the Lands cycle may fail to maintain optimal membrane composition, leading to functional compromise.^224,225^ These imbalances are often exacerbated by insulin resistance, a hallmark of the metabolic syndrome.^226,227^

Membrane remodelling is a dynamic and essential process that depends on this integrated lipid network. While the Lands cycle modulates existing phospholipids, the Kennedy pathway provides precursors for de novo phospholipid biosynthesis.^228^

Sphingolipids contribute to membrane microdomain organization and signaling, while fatty acid oxidation and lipophagy provide energy and remove damaged lipid components.^229,230^ Dysregulation of membrane remodelling has been implicated in several pathologies, including neurodegenerative diseases, cancer, liver dysfunction and cardiovascular disease.^231,232^

Maintaining membrane integrity is essential not only for maintaining compartmentalization and selective permeability, but also for anchoring proteins, generating signals, and regulating membrane fluidity.^233,234^ The enormous lipid diversity in cellular membranes—over a thousand different species in mammals—arises from variations in headgroups and hydrophobic chains, including fatty acid length, branching and degree of unsaturation.^235,236^

This diversity allows fine-tuning of membrane properties such as fluidity, curvature and domain organization, which are critical for processes such as cell division, signaling and protein trafficking.^2,237^

In response to membrane stress, adaptive lipid modifications can occur, including changes in unsaturation patterns and recruitment of protective enzymes, to maintain the functional continuity of the lipid bilayer.^238,239^

Membrane proteins often contain intrinsically unstable regions that rely on the surrounding lipid environment for their proper activity and stability.^240,241^

Consequently, the dynamic interplay between lipid biosynthesis, remodelling and degradation is fundamental to cellular function and homeostasis. Understanding these metabolic interconnections provides a robust framework for the development of innovative therapeutic strategies targeting metabolic, inflammatory, neurodegenerative, and oncological diseases.

## Disease Implications

The Lands cycle is pivotal to the homeostasis of cell membranes, and its dysfunction is linked to a number of pathological conditions, including chronic inflammation, neurodegenerative diseases, cancer and liver diseases. Inadequate regulation of key enzymes in this cycle, such as PLA_2_ and acyltransferases, has been demonstrated to profoundly alter the lipid composition of membranes, impacting essential cellular processes such as signaling, response to oxidative stress and programmed cell death.^131,242^

In the context of inflammation, heightened activation of PLA_2_ leads to the release of arachidonic acid, a precursor of pro-inflammatory eicosanoids. This, in turn, amplifies the immune response and promotes the development of chronic inflammatory diseases. Furthermore, changes in the activity of LPCATs, particularly LPCAT1 and LPCAT3, modulate the incorporation of polyunsaturated fatty acids into membranes, thereby influencing the sensitivity of cells to ferroptosis—a type of cell death associated with lipid peroxidation.^9,87,131,243–245^

In neurodegenerative diseases such as Alzheimer’s and Parkinson’s, oxidative stress and chronic inflammation play a significant role in neuronal degeneration. The brain is particularly susceptible to oxidative damage due to its high oxygen consumption, low antioxidant capacity and high lipid content. Lipid peroxidation leads to loss of cell membrane integrity and increased permeability to ions such as calcium, exacerbating neuronal dysfunction.^176,246,247^

Dysregulation of the lipid cycle compromises lipid remodelling, impairs vesicular trafficking and contributes to the accumulation of misfolded proteins such as β-amyloid. In addition, changes in phosphatidylserine asymmetry are associated with mitochondrial dysfunction, exacerbating oxidative stress and promoting neuronal cell death.^247,248^ These processes are amplified by the activation of microglia and astrocytes, which produce pro-inflammatory mediators.^249^

Oxidative stress also plays a critical role in cardiovascular disease. Phospholipid peroxidation induces cell membrane instability in cardiac tissue, contributing to arrhythmias and ischemic events. Oxidation of LDL forms toxic particles (oxLDL) that trigger vascular inflammation and atherosclerosis.^250,251^

Imbalances in the control of oxidative stress, inflammatory homeostasis and lipid metabolism have profound implications for human pathophysiology, serving as hallmarks of several diseases and as potential biomarkers and promising therapeutic targets. The pathophysiological mechanisms involved in various clinical conditions are detailed below.^195,202,248^

The understanding of the molecular mechanisms involved in oxidative stress, inflammation and lipid remodelling has driven the development of innovative therapies. Antioxidants, inhibitors of inflammatory enzymes, modulators of nuclear receptors (such as PPARs) and compounds that interfere with redox signaling have shown potential in preclinical and clinical studies.^95,251^

Chronic inflammation and oxidative stress act synergistically in cancer to promote uncontrolled cell proliferation, immune evasion and tumor angiogenesis. Excessive generation of ROS amplifies mutations in cellular DNA and favors tumor progression.^176,246^

In the context of cancer, the overexpression of PLA_2_ and the dysregulation of LPCATs contribute to the reorganization of the plasma membrane. This, in turn, promotes increased proliferation, invasion, and resistance of tumor cells to apoptosis and chemotherapy. The LPCAT3–GPX4–iPLA_2_ axis has been identified as a critical regulator of the balance between iron-dependent cell repair and death. This finding suggests that these enzymes could be promising targets for new therapeutic strategies in resistant tumors. Dysfunctions in the liver’s Lands cycle have been demonstrated to compromise hepatocyte integrity, thereby exacerbating inflammatory processes and increasing susceptibility to drug-induced hepatotoxicity. It is therefore evident that pharmacological modulation of PLA_2_ and LPCATs is a potential approach that could be utilized to limit liver damage and promote tissue regeneration.^131,244,252,253^

In addition, lipid-based pharmacology—including endocannabinoid analogues, resolvins and lipoxins—represents a promising frontier in personalized medicine and may hold promise in the treatment of cardiovascular and oncological diseases.^202,246^

## Materials and Methods

The study took a multifaceted approach using two different software tools to analyze and evaluate the binding affinities of compounds to the active sites of liver enzymes. The powerful molecular docking capabilities of GOLD (Genetic Optimization for Ligand Docking) were used in conjunction with the DataWarrior (DW) software.^254,255^ This combination enabled molecular docking simulations to be performed that predicted the preferred orientation of the molecules in relation to the active sites of the enzymes under investigation.

The chemical obtained on the ChemBL database. DW was used to obtain parameters for the molecular properties of these compounds, and a parametrized library was generated at a suitable pH for later use by GOLD. Molecular Docking was carried out by GOLD, where 100 replicates per compound were made from a previously prepared library, the enzymes in the PDB (Protein Data Bank) databases. Both proteins already had reference ligands that were used during the tests to guide the results, which also required the use of the normalized score, which verifies the strength of the interaction in relation to the number of atoms in the ligands.^256,257^

The results were analyzed by DW using the piecewise linear potential (PLP) score, a parameter generated by GOLD to objectively quantify the binding affinities of the compounds. This algorithm quantitatively evaluates several factors, such as the structural complementarity of the compound with the target site and the interaction energy. This integrated approach provided valuable information on the interaction between the compounds and the active sites of the enzymes.^255,258^ All figures using these studies were generated using Pymol^258^ software.

Molecular dynamics (MD) simulations are performed using tools such as AMBER 21 to build models of cell membranes and study the dynamics of the target protein inserted into the membrane.^259^ In the context of protein simulations, the AMBER14SB force field was employed, while organic molecules were parametrized using ANTECHAMBER based on GAFF, with RESP charges derived from Gaussian16 at the HF/6–31G­(d) level. The models were positioned within a rectangular box containing TIP3P water, with a minimum distance of 15 Å maintained between the protein and the box boundary. In order to neutralize the system’s charge and achieve the desired ionic strength, the addition of Na+ and Cl- ions was necessary. Energy minimization of the docked complexes was conducted in four stages: (1) water molecules, (2) hydrogen atoms, (3) protein amino acid side chains, and (4) the entire system. Each stage was performed for 10,000 steps. System equilibration was conducted utilizing the NVT and NPT ensembles, with gradual heating over 2000 ps, regulated by the Langevin thermostat at 310 K. Production molecular dynamics were executed for 100 ns, subsequently accompanied by a range of analyses. The calculation of the root-mean-square deviation (RMSD) and the root-mean-square fluctuation (RMSF) of the protein was performed using CPPTRAJ, with Xmgrace utilized for the generation of the corresponding graphs.^259^

The protein–ligand binding free energy was evaluated using MM-GBSA methods, based on at least 500 conformations obtained from the molecular dynamics, to improve docking predictions by accounting for protein and molecule flexibility. These methods also facilitated the identification of the energetic contributions of specific amino acids at the active site that are involved in coordinating the molecules.

The results of the molecular dynamics simulations are analyzed using Root Mean Square Deviation (RMSD), Molecular Mechanics Generalized Born Surface Area (MM-GBSA). By combining these data with experimental IC_50_ values, it is possible to validate the computational predictions and guide the design of more effective compounds.^239,241,257^ This work proposes to integrate these tools and techniques to create a bioinformatics pipeline for protein structure analysis and mechanism assessment from literature data.

The Lands cycle is pivotal in preserving cell membrane homeostasis by regulating lipid composition, fluidity, signaling, and the adaptive response to environmental stimuli. Recent studies have emphasized the significance of precise regulation of PLA_2_ and LPCAT enzymes in maintaining membrane integrity and in mediating pathophysiological processes such as inflammation, oncogenesis, and oxidative stress responses.^21,260^

Dysregulation of the arachidonic acid cascade has been linked to heightened inflammatory responses, primarily due to increased PLA_2_ activity, which leads to the release of arachidonic acid and the subsequent production of eicosanoids.^87^ Modulation of LPCAT activity, particularly the LPCAT1 and LPCAT3 isoforms, also impacts the incorporation of PUFAs into membranes, influencing cellular susceptibility to ferroptosis, an iron-dependent form of cell death driven by lipid peroxidation.^261,262^

In the context of cancer, the overexpression of PLA_2_ and the subsequent alteration of LPCAT activity have been demonstrated to contribute to the remodelling of the plasma membrane. This, in turn, has been shown to support tumor cell proliferation, invasion, and resistance to apoptosis and chemotherapy.^263^ The LPCAT3–GPX4–iPLA_2_ axis has recently been identified as a key modulator of the balance between repair and ferroptosis, emphasizing its potential as a therapeutic target in treatment-resistant cancers.^262,264^

In hepatic tissue, disruptions in the Lands cycle compromise hepatocyte integrity, intensify inflammation, and exacerbate drug-induced liver injury.^265^ The pharmacological targeting of these enzymes is a strategy that is being explored as a means of attenuating liver damage and promoting regeneration.

Advancements in the fields of lipidomics and computational modeling have facilitated the identification of lipid signatures associated with specific diseases, thereby establishing the foundations for personalized medicine.^244^ Furthermore, environmental factors such as diet and exercise have been demonstrated to influence Lands cycle activity, thus offering potential nonpharmacological interventions for the prevention and management of chronic disease.^266^

Despite the growing body of knowledge in this field, significant gaps remain in our understanding, particularly with regard to the molecular regulation of PLA_2_ and LPCAT across different tissues and under physiological versus pathological conditions.^56,267^ The mechanisms that govern the specificity of enzymes involved in lipid remodelling, and how these vary between cell types and environmental contexts, remain insufficiently understood and require detailed biochemical and structural studies.^197^

The role of PLA_2_ in cancer is a contentious topic. A number of studies have been conducted which appear to demonstrate an association between its overexpression and tumor progression, as well as a poor prognosis. However, it should be noted that other studies have failed to confirm a direct causal link. This underscores the necessity for additional research to elucidate whether PLA_2_ functions as an initiator of malignancy or a secondary participant in tumor metabolism.^116,176,213^

Therapeutic modulation of PLA_2_ and LPCAT has been demonstrated to be a promising approach, particularly in the context of overcoming apoptosis resistance. However, achieving specificity without off-target effects remains a significant challenge. A more profound comprehension of these enzymes’ context-dependent functions is imperative for the development of safe and effective inhibitors or activators.^191,201^ Furthermore, emerging evidence suggests a role for phospholipid remodelling in neurodegenerative diseases such as Alzheimer’s and Parkinson’s. While there is evidence to suggest that altered PLA_2_ and LPCAT activity may be detrimental to synaptic plasticity and promote neuroinflammation, the extent of their contribution to disease progression remains to be elucidated.^89,165,166,223,247,248^

In the context of metabolic disorders, the relationship between the Lands cycle and systemic lipid homeostasis remains to be fully elucidated. Although phospholipid remodelling influences membrane composition and inflammation, the specific mechanisms connecting the Lands cycle to obesity, diabetes, and nonalcoholic fatty liver disease require further investigation.^166,194,225,250,251^

The subsequent critical step is the integration of the Lands cycle into broader metabolic and signaling networks. It is evident that lipid remodelling is intricately linked with various signaling pathways, including eicosanoid, oxidative stress, and immune responses. In order to comprehend these interactions, it is necessary to employ a systems biology approach.^95,195,195,202^

In order to address these knowledge gaps, future studies should give priority to longitudinal analyses in order to clarify the role of the Lands cycle in chronic disease progression. The investigation of tissue-specific modulation has the potential to facilitate the design of targeted therapies that exhibit minimal side effects. Furthermore, the exploration of interactions between the Lands cycle, gut microbiota, and systemic metabolism has the potential to yield novel strategies for the prevention and treatment of disease.^1^

The present research seeks to contribute to this field by employing in silico modeling and a review of the literature to investigate how selected NSAIDs may modulate key enzymes of the Lands cycle. This approach may inform the development of more precise, personalized cancer therapies. Further exploration of the molecular underpinnings and therapeutic potential of this metabolic axis has the potential to drive significant advancements in the treatment of cancer, neurodegenerative diseases, and metabolic disorders.

## Conclusions

The Lands cycle has emerged as a central biochemical process that goes beyond phospholipid remodelling and plays a crucial role in cellular homeostasis and disease pathogenesis. The key enzymes, PLA_2_ and LPCAT, exhibit complex structural and functional properties that influence membrane dynamics, cellular signaling and oxidative stress responses. Their catalytic mechanisms and subcellular localization add layers of regulation that link the Lands cycle to energy metabolism and cellular stress pathways.

Detailed examination of the structural features of these enzymes has revealed the presence of highly sophisticated catalytic mechanisms. For example, cPLA_2_ uses a Ser-Asp dyad mechanism in its active site, where Ser228, supported by Asp549, acts as a nucleophile to facilitate the hydrolysis of the sn-2 ester bond in phospholipids. This chemical specificity is important for the selective release of fatty acids such as arachidonic acid, a precursor of pro-inflammatory eicosanoids. Conversely, LPCAT utilizes an acetyl-CoA transfer mechanism in which the His351 residue plays a pivotal role in catalysis, facilitating efficient reacylation of lysophospholipids. These mechanistic differences provide a unique opportunity for the development of selective inhibitors.

The subcellular compartmentalization of these enzymes introduces another layer of regulation, integrating the Lands cycle with energy metabolism and the cellular stress response. Studies using cell fractionation and confocal microscopy have shown that cPLA_2_ translocate to the perinuclear and endoplasmic reticulum membranes in response to increases in intracellular Ca^2+^. In contrast, different LPCAT isoforms show distinct distributions between the endoplasmic reticulum and lipid droplets. This differential localization not only optimizes substrate access but also coordinates the Lands cycle with de novo phospholipid biosynthesis and fatty acid β-oxidation.

Recent advances in analytical techniques, including high-resolution mass spectrometry and cryo-electron microscopy, have provided unprecedented insights into the molecular dynamics of phospholipid remodelling. These approaches have deepened our understanding of lipid compositional changes in pathological conditions such as cancer and neurodegenerative diseases, while facilitating the rational design of selective inhibitors. However, significant knowledge gaps remain, particularly regarding the integration of the Lands cycle with other pathways, such as autophagy and ER stress responses.

The therapeutic potential of targeting the Lands cycle is considerable, but challenges remain in translating this knowledge into clinical applications. While preclinical studies have demonstrated the efficacy of PLA_2_ inhibitors in reducing the progression of atherosclerosis, functional redundancy between enzyme isoforms and systemic effects of cycle modulation complicate drug development. Novel strategies, including allosteric inhibitors and genome editing approaches, may offer more precise and effective interventions in the future.

However, the translation of these findings into clinical practice has been hampered by the functional redundancy between different enzyme isoforms and the pleiotropic effects of Lands cycle modulation. The development of new strategies, including the creation of allosteric inhibitors and genome editing techniques, represent promising avenues to address these challenges.

As our understanding of the Lands cycle continues to expand, its importance at the intersection of structural biochemistry, cell biology and translational medicine is becoming increasingly apparent. This pathway holds great promise for the development of targeted therapies, with potential applications ranging from inflammatory diseases to oncology. Future research in this area will not only refine our understanding of lipid metabolism but also pave the way for groundbreaking advances in molecular medicine.

## Abbreviations

(4-HNE): 4-hydroxynonenal
(AA): arachidonic acid
(Ca^2+^): calcium ions
(COPD): chronic obstructive pulmonary disease
(CoASH): CoA group
(COX-1): cyclooxygenase-1
(COX-2): cyclooxygenase-2
(DW): DataWarrior
(DEP): diethyl phosphate
(diHETEs): dihydroxyeicosatrienoic acids
(DPPC): dipalmitoylphosphatidylcholine
(DHA): docosahexaenoic acid
(DILI): drug-induced liver injury
(EPA): eicosapentaenoic acid
(EETs): epoxyeicosatrienoic acids
(Fe^3+^): ferric iron
(Fe^2+^): free ferrous ions
(GPX4): glutathione peroxidase 4
(GOLD): genetic optimization for ligand docking
(PAFR): G-protein-coupled platelet-activating factor receptor
(His): histidine
(IHE): indomethacin heptyl ester
(LOX): lipoxygenases
(LXs): lipoxins
(LTs): leukotrienes
(Lp-PLA_2_): lipoprotein-associated phospholipase A_2_
(LPCAT): lysophospholipid acyltransferase
(LPC): lysophosphatidylcholine
(LPL): lysophospholipids
(MDA): malondialdehyde
(MBOAT): membrane-bound *O*-acyltransferase
(ACSM/ACSL): medium/long-chain acyl-CoA synthetase
(MD): molecular dynamics
(NSAIDs): nonsteroidal anti-inflammatory drugs
(oxLDL): oxidized low-density lipoprotein
(OXY-FA): oxidized polyunsaturated fatty acids
(Prdx6): peroxiredoxin 6
(PA): phosphatidic acid
(PC): phosphatidylcholine
(PE): phosphatidylethanolamine
(PI): phosphatidylinositol
(PS): phosphatidylserine
(PLA_2_): phospholipase A_2_
(cPLA_2_): cytosolic PLA_2_
(sPLA_2_): secreted PLA_2_
(iPLA_2_): calcium-independent PLA_2_
(PAF-AH): platelet-activating factor acetyl hydrolase
(PAF): platelet-activating factor
(PLB): phospholipase B
(PLP): piecewise linear potential
(PUFAs): polyunsaturated fatty acids
(PDB): Protein Data Bank
(ROS): reactive oxygen species
(GSH): reduced glutathione
(GSSG): oxidized glutathione
(ROH): reduction of hydroperoxides (ROOH) to their corresponding alcohols
(RMSD): root-mean-square deviation
(RMSF): root-mean-square fluctuation
(TLRs): toll-like receptors
